# A realistic benchmark for differential abundance testing and confounder adjustment in human microbiome studies

**DOI:** 10.1186/s13059-024-03390-9

**Published:** 2024-09-25

**Authors:** Jakob Wirbel, Morgan Essex, Sofia Kirke Forslund, Georg Zeller

**Affiliations:** 1 Structural and Computational Biology Unit (SCB), European Molecular Biology Laboratory (EMBL), Heidelberg, Germany; 2 Experimental and Clinical Research Center (ECRC), a cooperation of the Max-Delbrück Center and Charité–Universitätsmedizin, Berlin, Germany; 3 Max-Delbrück Center for Molecular Medicine in the Helmholtz Association (MDC), Berlin, Germany; 4 Charité–Universitätsmedizin Berlin (a corporate member of Freie Universität Berlin and Humboldt–Universität zu Berlin), Berlin, Germany; 5 German Center for Cardiovascular Research (DZHK), Partner Site Berlin, Berlin, Germany; 6 Center for Infectious Diseases (LUCID), Leiden University, Leiden University Medical Center (LUMC), Leiden, Netherlands; 7 Center for Microbiome Analyses and Therapeutics (CMAT), Leiden University Medical Center, Leiden, Netherlands

**Keywords:** Microbiome, Benchmark, Metagenomics, Differential abundance, Confounding

## Abstract

**Background:**

In microbiome disease association studies, it is a fundamental task to test which microbes differ in their abundance between groups. Yet, consensus on suitable or optimal statistical methods for differential abundance testing is lacking, and it remains unexplored how these cope with confounding. Previous differential abundance benchmarks relying on simulated datasets did not quantitatively evaluate the similarity to real data, which undermines their recommendations.

**Results:**

Our simulation framework implants calibrated signals into real taxonomic profiles, including signals mimicking confounders. Using several whole meta-genome and 16S rRNA gene amplicon datasets, we validate that our simulated data resembles real data from disease association studies much more than in previous benchmarks*.* With extensively parametrized simulations, we benchmark the performance of nineteen differential abundance methods and further evaluate the best ones on confounded simulations. Only classic statistical methods (linear models, the Wilcoxon test, *t*-test), *limma*, and *fastANCOM* properly control false discoveries at relatively high sensitivity. When additionally considering confounders, these issues are exacerbated, but we find that adjusted differential abundance testing can effectively mitigate them. In a large cardiometabolic disease dataset, we showcase that failure to account for covariates such as medication causes spurious association in real-world applications.

**Conclusions:**

Tight error control is critical for microbiome association studies. The unsatisfactory performance of many differential abundance methods and the persistent danger of unchecked confounding suggest these contribute to a lack of reproducibility among such studies. We have open-sourced our simulation and benchmarking software to foster a much-needed consolidation of statistical methodology for microbiome research.

**Supplementary Information:**

The online version contains supplementary material available at 10.1186/s13059-024-03390-9.

## Background

The human gut microbiome is increasingly understood to play critical roles in host physiology and immunity and thus mined for health and disease biomarkers. Taxonomic composition of gut microbial communities is highly variable between individuals [1, 2], yet clinical microbiome association studies strive to overcome inter-individual differences to identify microbial features that differ significantly between groups of individuals. Numerous diseases have been linked to specific microbes (including but not limited to inflammatory bowel diseases [3, 4], gastrointestinal cancers [5, 6], and cardiometabolic diseases [7]), typically by independently testing bacterial taxa for significant differential abundance (DA) between disease and control groups. DA methods loosely fall into three broad categories: (a) classical statistical methods, (b) methods adapted from (bulk) RNA-Seq analysis, or (c) methods developed specifically for microbiome data.

While many studies have reported significant microbiome disease associations, some meta-analyses and cross-disease comparisons have suggested many of them to be unspecific or confounded [8–10], i.e., attributable to other factors. For example, it is currently estimated that oral medication, stool quality, geography, and alcohol consumption collectively account for nearly 20% of the variance in taxonomic composition of gut microbiota [11, 12], yet these lifestyle factors often differ systematically between healthy and diseased populations being compared [13, 14]. As a well-known example, two different studies reported associations between type 2 diabetes (T2D) and certain gut taxa which were later identified as a metformin response in a subset of T2D patients [8]. Furthermore, technical or batch effects resulting from non-standardized experimental protocols are prevalent in metagenomic studies [15, 16] and can outweigh biological differences of interest [6, 17, 18].

Although unique characteristics of microbiome sequencing data—either whole meta-genome sequencing (WGS) or 16S ribosomal RNA amplicon sequencing (16S)—are well-described by now [19–21], there is no consensus about the most appropriate DA procedures in the literature [22–29]. In principle, this is the purpose of benchmarking studies, which typically use parametric methods to simulate differentially abundant features under a ground truth for performance evaluation. Yet, for benchmarking conclusions to translate to real-world applications, it is essential that simulated data recreate key characteristics of experimental data, which is why resampling techniques have also been used for benchmarking [26, 30].

Thus, on the more fundamental question of how best to simulate microbiome data, there is no consensus either. Existing simulation models have not been thoroughly validated on their ability to reproduce and resemble real experimental data (“biological realism”), nor has the impact of this property on downstream benchmarking applications been properly evaluated. Furthermore, despite a growing awareness of confounders in microbiome association studies, no evaluation of DA methods has meaningfully addressed this topic before.

To address these issues, here we propose a simulation technique using in silico spike-ins into real data, causing specific taxa to differ in abundance and/or prevalence between two groups (imitating a case–control design). In an extension of these simulations, we include confounding covariates with effect sizes resembling those in real studies. We quantitatively assess the degree to which parametric simulations employed in previous DA benchmarks lack biological realism, and show that the choice of simulation framework can explain divergent recommendations regarding DA methods. Based on our more realistic simulations, we perform a comprehensive benchmarking study of widely used DA methods and observe that many of these either do not properly control false positives or exhibit low sensitivity to detect true positive spike-ins. These issues are exacerbated under confounded conditions, but can largely be mitigated using the subset of methods which allows adjustment for a covariate. Finally, we explore the merits of confounder-adjusted DA testing on a large clinical dataset.

## Results

### Assessment of biological realism for parametrically-simulated taxonomic profiles

**Fig. 1:**
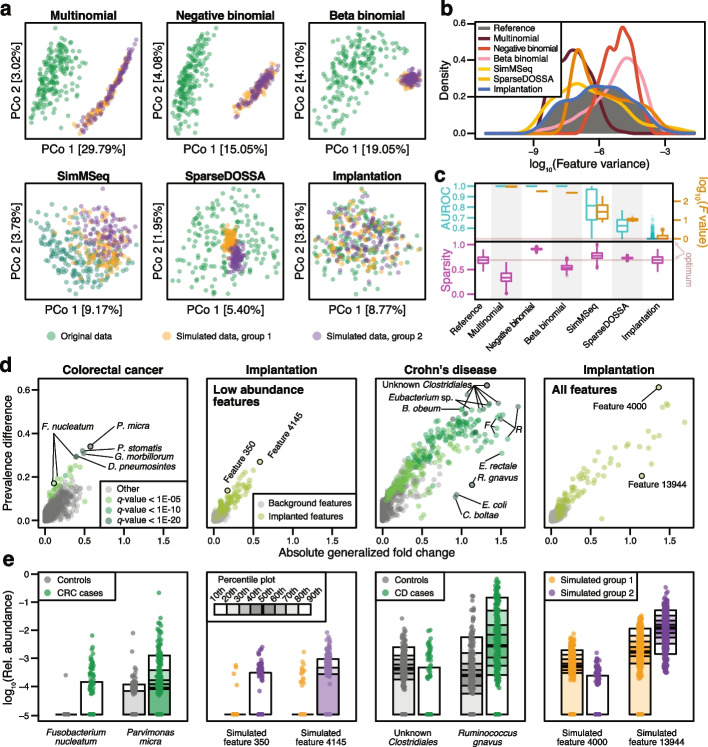
Signal implantation, but not parametric simulations, can reproduce key characteristics of metagenomic data and realistic disease effects.** a** Principal coordinate projections on log-Euclidean distances for real samples (from Zeevi et al. [33], which served as a baseline data set) and representative samples of data simulated in a case–control setting (groups 1 and 2) using different simulation frameworks or signal implantation. For each method, the results from a single repetition and a fixed effect size are shown (abundance scaling factor of 2 with an additional prevalence shift of 0.2 in our simulations, see the “Methods” section and Additional File 1: Fig. S4 for the complete parameter space). **b** Distributions of log-transformed feature variances shown for the real and simulated data from **a**. **c** The area under the receiver operating curve (AUROC) values from machine learning models (see the “Methods” section) to distinguish between real and simulated samples are shown across all simulated data sets in cyan. As complementary information, the log-transformed F values from PERMANOVA tests are shown in brown. Sparsity (fraction of taxa with zero abundance in a sample) is shown below in magenta. Boxes denote the interquartile range (IQR) across all values with the median as a thick black line and the whiskers extending up to the most extreme points within 1.5-fold IQR. **d** The absolute generalized fold change [6] and the absolute difference in prevalence across groups is shown for all features in colorectal cancer (CRC) and Crohn’s disease (CD). As a comparison, the same values are displayed for two data sets simulated using signal implantation (abundance scaling factor of 2, prevalence shift of 0.2), with implantations either into any feature or only low-abundance features (see the “Methods” section). Well-described disease-associated features are highlighted (*F*: *Faecalibacterium*, *R: Ruminococcus*) and selected bacterial taxa and simulated features are shown as percentile plot in **e**

As a first step, we aimed to explicitly evaluate how data generated from previous simulation frameworks compared to real metagenomic data. To do so, we simulated case–control taxonomic profiles using the source code employed in previous benchmarks [23, 25, 26, 31, 32], whereby datasets were repeatedly generated with differentially abundant features of varying effect sizes (see the “Methods” section) and compared these to the real input data. Simulation parameters were estimated in each case from the same baseline dataset of healthy adults, analyzed by shotgun metagenomic sequencing (Zeevi WGS [33]). We observed the data simulated with every one of the previously used simulation models to be very different from real data as was apparent from principal coordinate analysis (see Fig. 1a) and from large discrepancies between the distribution of feature variances and sparsity (see Fig. 1b, Additional File 1: Fig. S1–2, also for other baseline datasets). Similarly, we observed the mean–variance relationships of many simulated features to fall outside the range of the real reference data, especially for the multinomial and negative binomial simulations (see Additional File 1: Fig. S3a). Finally, we trained machine learning classifiers to distinguish between real and simulated samples (see the “Methods” section for implementation details), which was possible with almost perfect accuracy in nearly all cases, except for data generated from sparseDOSSA [31] (Fig. 1c, Additional File 1: Fig. S2). This classification attempt was motivated by the fact that machine learning, commonly employed in association studies to detect biomarkers, is highly sensitive to subtle differences between groups that may remain undetected by ordination-based analyses [10]. Overall, all of the assessed simulation frameworks produced unrealistic metagenomic data.

### Signal implantation yields realistic taxonomic profiles for benchmarking

To devise a more realistic simulation framework, we opted to manipulate real baseline data as little as possible by implanting a known signal with pre-defined effect size into a small number of features using randomly selected groups (similar to but distinct from the approaches of Jonsson et al. [30] and Thorsen et al. [34], see the “Methods” section and Additional File 2: Table S1). As a baseline dataset, we chose the same study population consisting of healthy adults (Zeevi WGS [33]), into which we repeatedly implanted a signal of differential abundance by multiplying the counts in one group with a constant (abundance scaling) and/or by shuffling a certain percentage of non-zero entries across groups (prevalence shift, see the “Methods” section). The main advantage of this proposed signal implantation approach is that it generates a clearly defined ground truth of DA features (like parametric methods) while retaining key characteristics of real data (a strength of resampling-based methods). In particular, feature variance distributions and sparsity were preserved (see Fig. 1b, Additional File 1: Fig. S1–2), as were the mean–variance relationships present in the real reference data (see Additional File 1: Fig. S3a), except at the most extreme effect sizes (see Additional File 1: Fig. S3b). Consequently, neither principal coordinate analysis (Fig. 1a), nor machine learning classification could distinguish our simulated from the real reference data (Fig. 1c, Additional File 1: Fig. S2). We observed these trends across all baseline datasets that we used for implantation (see Additional File 1: Fig. S1–3, see also the “Methods” section for details about included datasets).

### Implanted DA features are similar to real-world disease effects

To ensure our implanted DA features were comparable to those observed in real microbiome data in terms of their effect sizes, we focused on two diseases with well-established microbiome alterations, namely colorectal cancer (CRC) [5, 6] and Crohn’s disease (CD) [3, 4]. In two separate meta-analyses (see the “Methods” section), we calculated generalized fold changes as well as the differences in prevalence between controls and the respective cases for each microbial feature (see Fig. 1d, e). The effect sizes in CRC were generally found to be much lower than in CD, consistent with machine learning results in both diseases (mean AUROC for case–control classification: 0.92 in CD and 0.81 in CRC, see ref [10]). For instance, the well-described CRC marker *Fusobacterium nucleatum* exhibits only moderately increased abundance in CRC, but strongly increased prevalence. This observation, generalizable to many other established microbial disease biomarkers, motivated the inclusion of the prevalence shift as an additional type of effect size for the proposed implantation framework.

Depending on the type and strength of effect size used to implant DA features, the simulated datasets included effects that closely resembled those observed in the CRC and CD case–control datasets (Fig. 1de). In particular, simulated abundance shifts with a scaling factor of less than 10 were the most realistic and therefore used for subsequent analyses (Additional File 1: Fig. S4).

### Performance evaluation of differential abundance testing methods

To benchmark the performance of widely-used DA testing methods under verified realistic conditions, nineteen published DA tests were applied across all simulated datasets (see Additional File 2: Table S2 for a summary and the “Methods” section for implementation details). Different sample sizes were created by repeatedly selecting random subsets from each simulated group, and each test was applied to the exact same sets of samples (see the “Methods” section). For each method, we used the recommended normalization and also explored additional data preprocessing techniques such as rarefaction (see Additional File 1: Fig. S5).

The resulting *P* values were adjusted for multiple hypothesis testing with the Benjamini-Hochberg (BH) procedure to estimate recall and the method-specific false discovery rate (FDR), referred to as the estimated FDR. Using a cutoff of *P* < 0.05 to define discoveries (i.e., an estimated FDR of 5%), we also calculated the observed FDR from the ground truth of our benchmarking data as the fraction of false positives among all discoveries. Additionally, a receiver operating characteristic (ROC) analysis was carried out to evaluate how accurately the raw *P* values could distinguish between ground truth and background features. In the ideal case of *P* values for all ground truth features being smaller than for any of the background features, the area under the ROC curve (AUROC) will be one; for random *P* values an AUROC of 0.5 is expected.

**Fig. 2:**
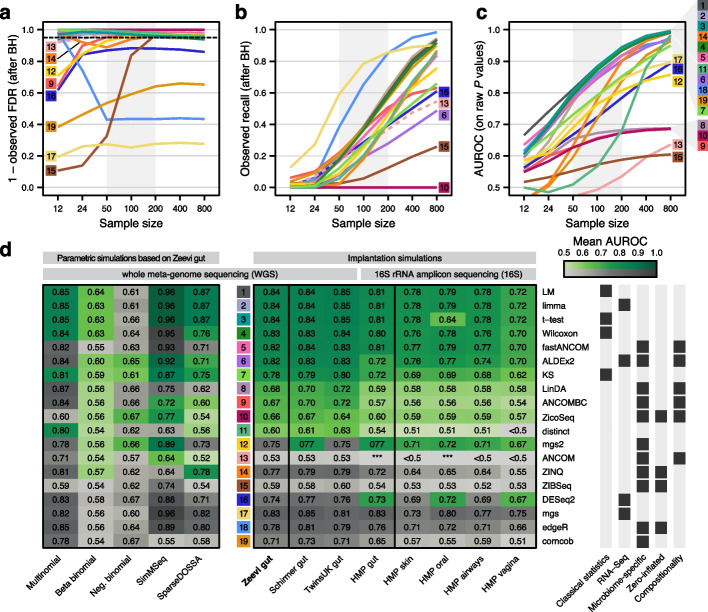
Performance evaluation of differential abundance testing methods and simulation strategies. For a signal implantation simulation with a single, moderate effect size combination (abundance scaling factor of 2, prevalence shift of 0.2, all features eligible for implantation), **a** the mean observed FDR and **b** the mean observed recall (calculated after Benjamini-Hochberg (BH) correction of raw *P* values) are shown for all included DA test methods across different sample sizes (see the “Methods” section). Additionally, mean AUROC values for differentiating between implanted and background features (calculated from raw *P* values) are shown in **c**. The nominally expected value of a 5% FDR is indicated by a dotted black line in panel **a**. Since ANCOM does not return *P* values (see the “Methods” section), observed FDR and recall were based on the recommended cutoffs (without adjustment) and therefore highlighted by dashed lines. Marginal annotations of method ranks correspond to the ranking based on AUROC values, with methods without sufficient FDR control ranked last (see panel **d**). **d** The mean AUROC values across all effect sizes and repetitions for the sample sizes 50, 100, and 200 (shaded area in **a**–**c**) are depicted in the heatmap for the different simulation strategies and baseline datasets, including non-gut human-associated microbiomes. The AUROC values of methods that exceeded a mean observed FDR of 10% in more than 10% of test settings (combination of effect and sample sizes) are shown in gray, whereas methods with sufficient FDR control are colored in shades of green. Methods with sufficient FDR control are ordered by their AUROC values on the Zeevi WGS gut dataset. For some simulations, the mean AUROC values were below 0.50 (indicated by < 0.5) or did not produce results in the allotted time (48 h for each combination of effect size, repetition, and sample size variation; indicated by stars)

We found that for several methods the observed FDR (i.e., the actual proportion of false positives) far exceeded the estimated FDR (i.e., the expected proportion of false positives based on the method’s assumptions, adjusted to 5%). This was especially true for sample sizes under 200 (displayed for a single representative effect size in Fig. 2a–c, see Additional File 1: Fig. S6 for other effect sizes). In the most extreme case, and in line with previous reports [23, 25], the *fitZig* method from metagenomeSeq (*mgs*) displayed an observed FDR of 80% (i.e., only 20% of features identified as significantly differentially abundant between groups were true in silico spike-ins). This behavior was observed across many sample and effect sizes (see Additional File 1: Fig. S6). To explore whether this issue was due to the BH FDR estimation procedure, we additionally applied the more conservative Benjamini-Yekutieli (BY) method and found the observed FDR to decrease, albeit at a loss of sensitivity (see Additional File 1: Fig. S7). Nevertheless, for some methods, large discrepancies between observed and estimated FDR were recorded under both procedures (see Additional File 1: Fig. S7), indicative of method-inherent lack of type I error control and in line with previous reports [26].

Of those DA methods for which the estimated FDR (resulting from BH correction) did not strongly deviate from the observed FDR, many also exhibited comparably low observed recall or AUROC values, indicating these methods were relatively insensitive. For example, the *ALDEx2* method exhibited very low recall across all repetitions (see Fig. 2b) and *ANCOM* resulted in a mean AUROC of 0.53 across all repetitions (see Fig. 2c, d), which is not significantly better than random guessing (*P* = 0.97, Wilcoxon test). In contrast, the methods with the highest AUROC values were classical statistical methods that were not designed for microbiome data (*Wilcoxon*, *t-test*, *limma*, and the linear model), with the exception of *fastANCOM*. These methods exhibited proper FDR control at comparably high sensitivity and were ranked at the top with remarkable consistency across other datasets (Fig. 2d), thereby emerging as reliable testing frameworks for the analysis of human-associated microbiome data.

### Impact of simulation approach and method normalization choices on benchmarking results

Our benchmarking results did, however, depend on the simulation method used for data generation. Negative binomial simulations in particular ranked *ZicoSeq* and *metagenomeSeq* as the top DA methods, almost entirely discordant with the rankings from other simulations, datasets, and biomes (see Additional File 1: Fig. S2d and Additional File 1: Fig. S8). For both the negative and beta binomial simulations, no DA method (including the respective top three) had a mean AUROC > 0.7 across sample sizes 50–200 (Fig. 2d). Interestingly, of the DA methods which had the overall poorest performance (i.e., exceeded 10% false discoveries across at least 10% of simulations), four methods assume one of these distributions in their models.

Most of the included DA methods use count tables as input and might perform method-specific normalization or variance-stabilizing procedures (see the “Methods” section). To explore the effect of rarefaction, one of the most commonly employed library size normalization techniques in microbiome data analysis, we additionally ran all methods with rarefied counts as input. On average, rarefaction led to a reduction of sensitivity (lower observed recall and lower AUROC values) across the majority of tested methods without improving the observed FDR (see Additional File 1: Fig. S5). For methods that do not model count data, we also explored other commonly used data preprocessing techniques, such as the total sum scaling (TSS) or centered log ratio (clr) transformation (see the “Methods” section for details). With TSS-log preprocessing, recall and AUROC of *limma*, the *t-test*, and the *LM* considerably improved, whereas the performance of the *Wilcoxon* test decreased after application of the clr and robust clr transformations (see Additional File 1: Fig. S5 and the “Methods” section).

While it has been argued by many microbiome researchers that sequencing produces compositional data [19], it is unclear from the literature to which extent computational approaches can successfully address it [35]. Advocates of such approaches argue that (unaddressed) compositionality leads to spurious associations as a consequence of the inherent correlation structure between features. To minimize unintended compositional correlation structures in our simulation framework, signal implantation alternated between groups by default (following Weiss et al. [25], see the “Methods” section). However, to assess DA method behavior in the presence of compositional effects, we also implanted signals into only one group in further simulations (see the “Methods” section), which led to abundance shifts in background features (i.e., spurious signals) in addition to the ground truth signals (see Additional File 1: Fig. S9). Under these conditions, the observed FDR increased for all tested methods with increasing effect sizes, with *ANCOM* and *ALDEx2* being the least affected (see Additional File 1: Fig. S9). Despite this, *fastANCOM* had an AUROC > 0.9 for the highest compositional effect sizes, noticeably outperforming other methods.

### DA method performance evaluated under confounded conditions

One major issue with the simple DA testing procedure outlined above is that it does not consider potentially confounding covariates (i.e., variables tracking the myriad ways case–control groups may differ in addition to disease status) as an important source of spurious associations. To mitigate this issue, association studies increasingly rely on stratification or multiple regression techniques to adjust for potential confounders in DA testing, but how well this works for microbiome studies has not yet been quantitatively evaluated.

**Fig. 3:**
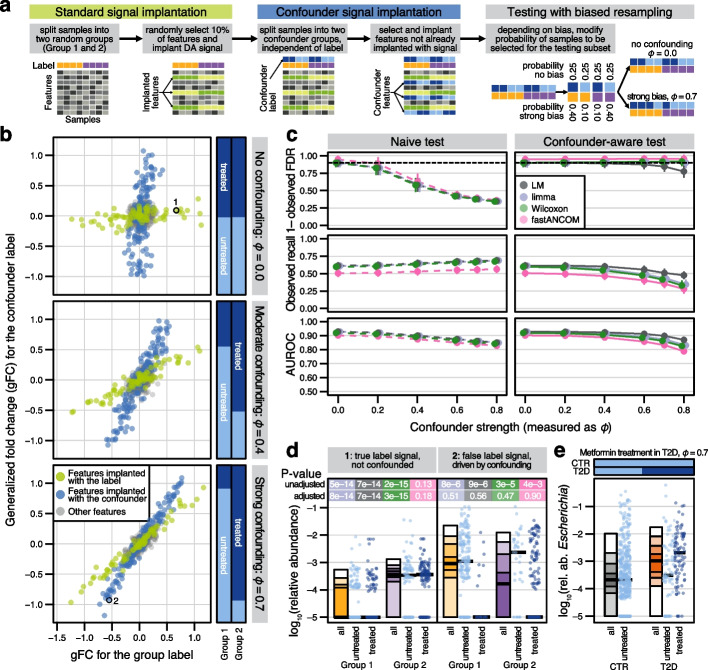
Loss of precision and recall under confounding can be alleviated by statistical adjustment.** a** Using a single dataset, DA features were independently implanted into a small proportion of taxa for both a main group label (as described above) and for an independent binary (confounder) label, imitating, e.g., disease and medication status labels, respectively. Subsets for DA testing were generated using a parameterized resampling technique such that the degree of association (measured by ϕ) between these two variables could be modified (i.e., deliberately biased). **b** Generalized fold change (gFC) calculated for the label is contrasted to the gFC calculated for differences between confounder values across all bacterial taxa (abundance scaling factor of 2, prevalence shift of 0.2, all features eligible for implantation, a single representative repeat shown). Bars at the right visualize the confounder strength by showing the proportion of confounder-positive samples in each group (with ϕ = 0 serving as unconfounded control). Main implanted features are highlighted in green and features implanted for the confounder label are in blue. **c** Mean observed FDR, observed recall (both calculated after BH-correction), and AUROC (on raw *P* values) for sample size 200 and the same effect sizes as shown in a) were computed for tested DA methods, using unadjusted and confounder-adjusted test configurations. Error bars indicate standard deviation around the mean for all repeats. **d** Simulated (log_10_ relative) abundances plotted by main and confounder labels (see Fig. 1 for definition of abundance quantiles), with both unadjusted and confounder-adjusted significance shown at the top, colored as in **c**. **e ***Escherichia* abundance appears naively associated with type 2 diabetes, yet is driven by metformin intake in a subset of diabetics (reproduced from Forslund et al.^*8*^)

To close this gap, we leveraged our simulation approach to mimic a simple but common scenario in a case–control study whereby a higher proportion of the disease group is taking medication than the control group. With the exception of antibiotics, the most commonly administered medication impacts a subset of microbial taxa [36]. Thus, we extended our previous simulations by implanting a second signal into a different (non-overlapping) subset of features, which divided our samples into four theoretical groups. Then, we implemented a biased resampling algorithm that generated data with an arbitrary correlation structure between our two simulated variables; the strength of this correlation could be modulated by adjusting the sampling probability (bias) for each group in order to simulate confounding (see Fig. 3a and the “Methods” section). We examined medicated group proportions in a real case–control study to ensure the realism of our chosen parameters (see Additional File 1: Fig. S10).

For the evaluation of DA methods, we focused on those found to control the FDR at concomitant high sensitivity in our previous benchmark (i.e., sufficient FDR control (see Fig. 2d), mean AUROC > 0.8, mean recall > 0.3), and which could additionally be adjusted for covariates (*limma*, the *LM*, *fastANCOM*, and the *Wilcoxon* test; an adjusted *t-test* would be implemented as a linear model with fixed effects, see Additional File 1: Fig. S11). To evaluate the effect of adjusting for known confounders on testing accuracy, we compared these methods in their naive (unadjusted) and adjusted configurations on our simulated data.

In the negative control setting (no confounding), unbiased sampling produced four balanced groups (which can occur, e.g., matched demographic factors like sex) and manifested as non-overlapping effects between the two simulated variables (see Fig. 3b). In contrast, larger bias values (representing, e.g., a higher prevalence of medication intake in the disease group) resulted in progressively overlapping signals (i.e., confounding), thereby making it difficult to attribute differential abundance in individual taxa to the respective grouping variables (i.e., to distinguish disease from medication effects, see Fig. 3b). In this confounded scenario, naive testing led to dramatically inflated observed FDR as confounder strength increased (observed FDR ~ 40% at moderate confounding with ϕ = 0.4, see Fig. 3c and Additional File 1: Fig. S12 for other datasets). Adjusted tests, however, generally maintained FDR control, although the performance of the *LM* weakened under extreme confounding (implemented as a linear mixed-effect model, *LMEM*, see the “Methods” section and Additional File 1: Fig. S11). The *LM* nonetheless exhibited the highest overall AUROC, results which were consistently observed in other baseline datasets and at varying proportions of implanted confounder-associated features even if the theoretical modeling assumptions were seldom met (see Additional File 1: Fig. S13).

To illustrate method behavior at the level of individual simulated feature abundances, we selected true and spurious signals from the ground truth, under a control setting and under strong confounding, respectively (see Fig. 3b). In the control setting, only *fastANCOM* failed to identify the true DA feature (see Fig. 3d, panel 1), which was not entirely unexpected considering its lower overall sensitivity (see Fig. 3c). In contrast, when biased resampling produced an association between the two grouping variables, we observed a clear false positive driven by confounding, as could be diagnosed from greatly decreased statistical significance when comparing the adjusted with the naive DA test results (see Fig. 3d, panel 2).

Importantly, our bias parameterization (confounder strength) in these simulations tracked well with phi coefficients calculated on real data (see Additional File 1: Fig. S10), and our implanted effect sizes resembled the actual effects observed for metformin treatment in T2D patients (see Fig. 3e).

To simulate confounding due to study heterogeneity as typically encountered in meta-analyses, we generated additional benchmarking data by combining samples from pairs of real study populations in healthy adults [33, 37, 38] before signal implantation, using study origin as the covariate in our biased resampling approach (see the “Methods” section and Additional File 1: Fig. S14–15). These benchmarks not only confirmed that a loss of FDR control under confounding affected all naive DA tests evaluated, but also that confounders acting more broadly than those simulated in Fig. 3 could be effectively addressed by adjusted tests. Overall, our results suggest that measured confounders (as long as ϕ < 0.8, see Fig. 3e) can be effectively controlled or adjusted for during DA testing.

### Discerning robust from confounded associations *in real* datasets

**Fig. 4:**
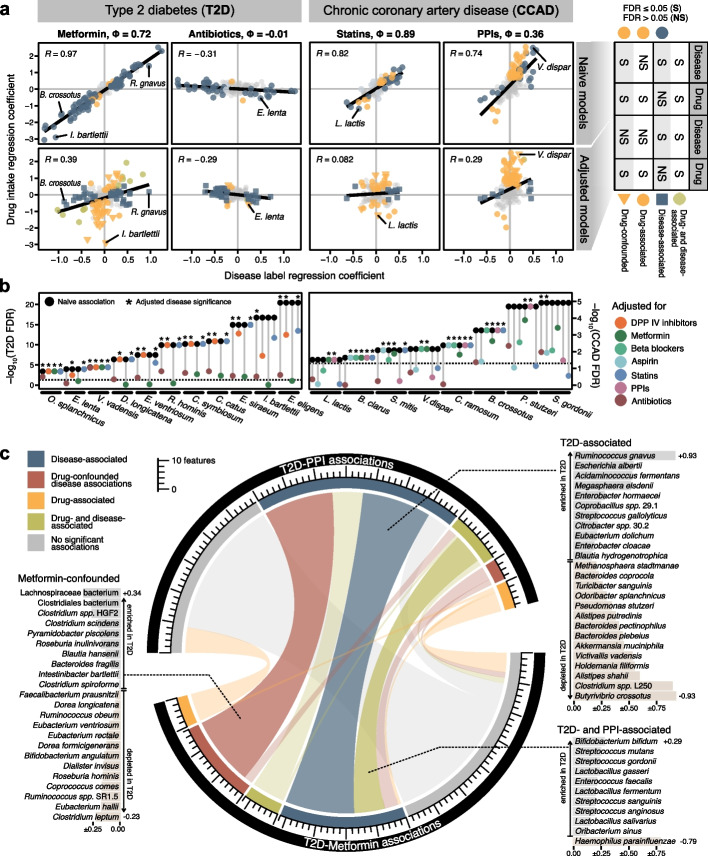
Linear models are capable of disentangling drug- and disease-associated microbial features.** a** Regression coefficients from a subset of disease-drug combinations comparing naive linear models to adjusted mixed-effect models for all bacterial taxa. Adjusted models included a second term (either drug intake or disease status for the *x*- and *y*-axes, respectively) as a random effect, which diminished the strong linear dependence between naive model coefficients (shown). When the significance of each term was compared between the naive and adjusted models (see the “Methods” section) drug-specific or confounded effects were revealed in some features.** b** Exemplary subset of features displaying either the largest number of significant disease associations across different drug-adjusted models or the largest reductions in disease coefficient significance upon adjustment (i.e., most confounded). **c** Comparison of feature classifications (see the “Methods” section) from the metformin- and PPI-adjusted disease association models across all bacterial taxa. Integrating information across models restricts disease associations to a more robust subset and reveals drug-confounded associations. Adjusted T2D regression coefficients are shown in light gray or light brown bars behind species names (indicating enrichment in T2D or control group, respectively)

To further explore the consequences of naive association testing compared to adjusted tests in real data, we applied both approaches to gut metagenomic samples from cardiometabolic disease patients in the MetaCardis cohort [14, 39, 40]. The strongest confounding potential was seen for chronic coronary artery disease (CCAD) and commonly-indicated medications taken by a large fraction of these patients, especially statins and aspirin (ϕ = 0.89 and ϕ = 0.9, respectively), as well as type 2 diabetes (T2D) and metformin (ϕ = 0.72, see Additional File 1: Fig. S10). Four linear models were built for each disease-drug combination (naively testing for disease or drug associations, respectively, and corresponding adjusted models for each) across all species-level taxonomic abundances. The resulting coefficients and *P* values were used to classify each feature with respect to both drug and disease associations (see the “Methods” section). As expected, large phi coefficients manifested as a strong linear relationship between naive *LM* coefficients, confirming that confounding can be diagnosed from such models (compare Figs. 3b and 4a). Importantly, the inclusion of random effects in the adjusted models helped to disentangle these overlapping effects and exposed drug- or disease-specificity in numerous individual associations (Fig. 4a).

In contrast to CCAD (and other diseases), T2D exhibited larger, more significant naive associations with more taxa (Fig. 4b). A little less than half of these were confounded by metformin treatment, as identified by the loss of a significant association with T2D and retention of a significant association with metformin in the adjusted models (Fig. 4b and the “Methods” section). Adjusting for antibiotic intake, on the other hand, did not significantly reduce the number of T2D-associated taxa, but generally reduced their coefficient size and significance. CCAD-associated taxa were sensitive to adjustment by multiple different drugs, including antibiotics, probably reflecting the complexity and variation typically seen in disease association studies where medication records are thoroughly analyzed. In general, confounder-adjusted linear models effectively helped to disentangle disease- and drug-associated taxa (evident from a reduced number of significant disease associations), consistent with their demonstrated ability to distinguish true positives from confounder positives in our simulation benchmarks (see Fig. 3c).

Metformin and proton pump inhibitors (PPIs) were among the largest drug effects observed in our analysis. Whereas most metformin-associated taxa were also naively T2D-associated, most PPI-associated taxa were not. This is in line with previous reports demonstrating metformin intake to correlate with T2D disease severity [8, 14, 41], and PPI-associated gut microbiota changes as a disease-independent presence of mainly oral commensals [42, 43] (consistent with the drugs’ mechanism of action). To further tease out disease-associated taxa, we cross-referenced the feature classifications we obtained from our models across different drugs as a robustness analysis. For example, PPI-adjusted models resulted in disease associations, several of which were metformin-confounded. By integrating information across both sets of confounder-adjusted models, we could uncover a more robust subset of disease-associated taxa (see Fig. 4c). Taken together, this analysis suggests that linear mixed-effect models are an effective and versatile method to improve the robustness of findings in association studies in which medication records are available*.*

## Discussion

Clinical microbiome research typically involves differential abundance testing to detect associations between host phenotypes, such as human diseases or responses to treatment [44], and individual microbial features in high-throughput. Numerous DA methods have been developed, encompassing a broad range of assumptions and hypotheses, but their performance has remained controversial, several previous benchmarking studies notwithstanding [22–27]. Based on our results, we argue that the lack of statistical consensus regarding optimal DA testing procedures for single studies is partially explained by the unvalidated and often unrealistic parametric simulations of microbiome data used as ground truth in previous benchmarks (see Additional File 2: Table S1). Here, we addressed this by implanting minimal, biologically-motivated modifications into real taxonomic profiles. Additionally, we extended our simulation framework to incorporate effects resembling confounders frequently encountered in microbiome studies. Although our strategy may lack the theoretical appeals of a parametric mathematical model, we empirically verified that our simulated taxonomic profiles were the only ones to retain essential metagenomic data properties and produce data that are virtually indistinguishable from real samples. Furthermore, our framework still provides the flexibility to specify the effect and sample sizes needed for an extensive evaluation against a ground truth, which was not the case in previous benchmarks built upon real datasets [26, 27, 45] (see Additional File 2: Table S1).

To make empirically guided recommendations on the suitability of DA methods, we performed a neutral benchmark based on our implantation framework that included many widely used DA methods. Evaluating each DA test on nearly one million simulated case–control data sets, we found that many methods yielded an excess of false positives, especially on smaller sample sizes (N < 100, see Fig. 1). Notable exceptions were classical statistical methods (the *Wilcoxon* test, *LMs*, and the *t-test*), *limma*, and *fastANCOM*, all of which produced an observed FDR close to theoretical expectations while retaining high sensitivity across a range of sample and effect sizes, datasets, and human-associated microbiomes.

Unlike previous benchmarks [23, 30], our results suggest DA methods borrowed from RNA-seq analysis, with the exception of *limma*, perform poorly on taxonomic microbiome profiles (further discussed in Additional File 3: Notes S1 and S2). Surprisingly, we also found most methods developed specifically for microbiome data to have comparably low power and high false positive rates (with the exception of *fastANCOM*) across the range of dataset sizes most commonly seen in contemporary studies (see Fig. 2d). On a positive note, at least when applied to larger samples (*N* ≥ 200 per group), more DA tests (including both *ANCOM* and *ANCOM-BC*, *ZIBseq*, *ZINQ*, and *metagenomeSeq2*) controlled the FDR as expected. Overall, our findings strongly support the use of classical statistical methods or the recently developed *fastANCOM*, and suggest that many studies employing other methods may have reported a substantial fraction of spurious microbiome associations.

This inferential risk is further exacerbated by confounding factors, which are rarely adjusted for during differential abundance testing, if recorded at all. While medication can be an obvious confounder for disease associations [14], large cohort studies also identified various lifestyle and physiological parameters, for example, alcohol intake or stool quality, as additional sources of heterogeneity [11–13]. As a more straightforward and scalable alternative to matching for all potential confounders in resampled groups (as proposed previously [13]), we explored simpler statistical adjustments using simulated data that mimicked well-understood [8, 12–14, 36] confounders. Reassuringly, adjusting the respective DA tests restored unconfounded performance to a large extent.

Limitations of our work include a narrow focus on human-associated taxonomic profiles from cross-sectional study designs, which precludes generalization to non-human microbial communities and longitudinal study designs. Moreover, we only minimally explored confounding by differential sequencing depth in our simulations, which has been investigated in more detail elsewhere [32]. However, future benchmarks wishing to explore the compositional nature of microbiome data in depth could also use our framework to generate simulations with differential sampling fractions between the two groups (see the “Methods” section).

Another limitation is that our confounded simulations were restricted to examining the impact of a single binary variable. Yet, linear (mixed-effect) models are very flexible and computationally efficient tools, which can readily accommodate continuous covariates such as stool microbial load [35, 46] and account for repeated measurements [29]. Notably, most newer compositional methods we tested (i.e., *ZicoSeq * [32], *ANCOM-BC * [47], *LinDA * [48], and *fastANCOM * [49]) adjust for a derived bias correction factor in a similar linear regression framework, in contrast to earlier methods relying on pairwise log-ratios (*ANCOM * [50]) or permutations on transformed values (ALDEx2 [51]), both of which are prohibitively computationally intensive (see Additional File 1: Fig. S16). Although we did not explore logistic regression DA tests here, methods that combine the flexibility to adjust for covariates with the robustness of non-parametric tests were proposed recently [52].

In our benchmark, we implemented *LMEM*s from the *lmerTest* R package [53] used in some DA tools such as *MaAsLin2 * [54] or *SIAMCAT * [10]. Given its popularity, we verified that our *LM* and *LMEM P* values were identical to the defaults of the *MaAsLin2* package. In our analysis of drug effects in T2D, we demonstrated the importance of further integrating deconfounded information to reveal robust disease-associated feature subsets. Analogous logic may be found in the vibration-of-effects paradigm [55, 56] and the *metadeconfoundR * [14, 57] package, which screens for potential confounders before performing combinatorial nested model DA tests on naive and confounder-adjusted linear models to classify feature robustness.

Given the limiting prerequisite that covariates need to be recorded for explicit adjustment, more attention will have to be paid to metadata collection and sharing in clinical microbiome studies. This will also enable meta-analyses to achieve biological consensus on disease- and treatment-associated microbiome features [6, 9, 10] through adjusting for potential confounders in their association tests. Here we provided a foundation for the choice of statistical methodology, which may aid in devising high-throughput robustness checks needed in single studies, as well as meta-analyses.

## Conclusion

In our view, the unsatisfactory performance of a wide range of DA methods and the persistent danger of unchecked confounding in the literature warrant a community effort to develop and benchmark more robust methodology. To assist researchers in developing and validating new DA methods, or establishing further benchmarks, both our signal implantation framework and our benchmarking analysis were designed to be easily extensible and are available as open source code (see the “Methods” section). Ultimately, community-driven benchmarking efforts similar to DREAM challenges [58] or the Critical Assessment of Metagenome Interpretation (CAMI) [59] project could accelerate the much-needed consolidation of statistical methodology for microbiome research.

## Methods

The first part of this “Methods” section describes the design of our simulations and its comparison to previous approaches as well as real microbiome sequencing data. The code for generating the simulated data is made available in an R package called SIMBA (https://github.com/zellerlab/SIMBA).

The second part of this “Methods” section contains details on how various differential abundance testing methods (in combination with different preprocessing routines) were applied to the simulated data, and how their results were evaluated against the ground truth and visually compared using custom scripts provided through the BAMBI R project (https://github.com/zellerlab/BAMBI).

### Data selection and preprocessing

The dataset from Zeevi et al. [33] was used as a baseline for the simulations in the main text. We included only those samples that had been analyzed via whole metagenome sequencing (WGS) and removed samples analyzed by 16S ribosomal RNA amplicon sequencing (16S). Additionally, we used the datasets from Schirmer et al. [38] (WGS) and Xie et al. [37] (TwinsUK WGS) as independent baselines for the simulation framework evaluation and assessment of microbiome data properties (see Additional File 1: Fig. S1–3), and combined different combinations of two WGS datasets at a time to mimic study effects in a meta-analysis setting (see Additional File 1: Fig. S14–15). All three WGS datasets consist of human gut microbiome samples. To explore other human-associated microbiomes, we also ran SIMBA on the abundance tables from the HMP1 dataset [60, 61], which included samples from different body sites.

For the gut WGS datasets, raw data were downloaded from ENA and analyzed as described before [10]. In short, after preprocessing and removal of host contamination, taxonomic profiling was performed with mOTUs2 (v2.5 [62]). All input data tables were filtered within SIMBA for prevalence (at least 5% across the complete dataset) and abundance (maximum relative abundance across samples of at least 1e − 04). In the case of repeated samples per patient, we selected only the first time point for each patient.

Lastly, the MetaCardis dataset [14] was used to explore drug confounding in real data. We used the cell-count adjusted, quantitative mOTUs profiles and metadata; a pseudocount of 1 was applied to zero counts before log transformation. In the case of repeated samples per patient, we selected only the first time point for each patient.

For all datasets, see the “Availability of data and materials” section for information about the raw data.

#### Parametric methods for the simulation of metagenomic data

To simulate metagenomic data on the basis of parametric methods, the implementations described in previous differential abundance benchmarking efforts were adapted into SIMBA (re-using the authors’ original source code wherever possible) and are briefly summarized in Additional File 2: Table S1, as well as here:

- Both McMurdie and Holmes [23] and Weiss et al. [25] used multinomial-generated counts, but differed slightly in how they created differentially abundant features. If not indicated otherwise, results for multinomial simulations were based on the implementation from Weiss et al., since the resulting effect sizes were closer to real effects (see Additional File 1: Fig. S4).
- Hawinkel et al. [26] included two different univariate parametric simulations based on the negative binomial and the beta binomial, as well as the multivariate Dirichlet distributions, all of which were included in SIMBA. Their non-parametric and real data shuffling methods were excluded on the grounds that they lacked sufficient parameterization for downstream benchmarking. For the beta binomial and optionally the negative binomial distribution, the correlation structure between bacterial taxa was estimated using SPIEC-EASI [20] as in the original publication. Values generated from the Dirichlet distribution were converted into counts via a post-processing rounding step.
- The Bayesian semiparametric method from Yang and Chen [32] was reproduced in SIMBA via the SimMSeq function of the GUniFrac R package.
- Lastly, to simulate data as described in Ma et al. [31], SIMBA relied on the dedicated functions in the sparseDOSSA R package.

Differentially abundant features were introduced into each parametric simulation as described in the respective original publications. For the multinomial simulations from McMurdie and Holmes as well as for the sparseDOSSA approach, features were scaled in abundance after the simulation was completed. In the case of the other simulation methods, the underlying parameters were adjusted with a scaling factor before the simulation. A range of effect sizes (abundances scaled by multipliers of 1, 1.25, 1.5, 2, 5, 10, and 20) was explored and for each effect size, a total of 20 repetitions was simulated (for each simulation method). At an abundance scaling factor of 1, no effects were introduced into the data and therefore those repeats served as internal negative controls. Simulation method implementations can be found in the respective helper_xxx.R files in SIMBA, and scripts to automate data generation on a SLURM cluster are stored in the create_simulations folder of the BAMBI repository.

### Implantation framework for the realistic simulation of microbiome data

To create benchmarking datasets without a parametric model, we implemented a novel simulation framework referred to as signal implantation (*helper_resampling.R* in SIMBA). In each simulated repetition, the original samples were randomly split into two groups, and 10% of features were randomly selected to become differentially abundant between the groups. Effects were implanted both via scaling abundances (using the same effect sizes as the parametric simulations) and by shifting prevalences (0.0, 0.1, 0.2, and 0.3).

For the abundance scaling, count values in one group were multiplied with a scaling factor to increase the abundances. The prevalence shifts were implemented by identifying non-zero counts in one group and randomly exchanging a specific percentage of those with occurrences of zero abundances in the other group (if possible), thereby creating a difference in prevalence across the groups with respect to the selected feature. Per default, the implantation of signals alternated between the two groups in order to prevent a systematic difference in total count number across groups (inspired by the considerations in Weiss et al. [25]). For each combination of effect sizes (abundance scaling and prevalence shift), 100 repetitions were simulated for the dataset from Zeevi et al. and 20 repetitions for all other datasets.

### Alternate configurations explored with the implantation framework

Knowing that some diseases preferentially associate with high- or low-abundance taxa (as in, e.g., colorectal cancer, see Fig. 1d, e), we additionally explored criteria to determine the set of features eligible for signal implantation: namely, *all*—all taxa were equally likely to be selected to carry a signal, or *low*—only low abundance features (the 75th percentile across all samples not exceeding 0). Other criteria we explored yielded unrealistic effect sizes (see Additional File 1: Fig. S4), and were not pursued further.

As a last step, the resulting generalized fold change [6] between the groups for all implanted features was recorded. Features with a fold change lower than 0.001 (resulting mostly from low-prevalence features being selected for implantation) were rejected and not recorded as implanted signals (this resulted in the removal of zero to three features across all repetitions of each simulated effect size, on average, depending on effect size). The reverse situation (i.e., background features with fold change values higher than the implanted features) was common for very low effect sizes, but otherwise, we generally observed a strong separation for background and implanted features as measured by AUROC (see Additional File 1: Fig. S4).

To generate simulations that mimic compositional effects (see Additional File 1: Fig. S9), the signal implantation was modified such that signals were implanted into only one group (i.e., not alternating between groups as per SIMBA default). Specifically, the balanced parameter was changed to *FALSE* in the *create.simulations* function. Lastly, the number of counts for each sample was scaled down to the original value of the unaltered sample by rarefaction using the *vegan* R package [63]. This last rarefaction step could be skipped in future benchmarks to create groups with different library sizes.

### Reality assessment for simulated data

To evaluate how well simulated metagenomic data approximated real data, for each “group” in a simulation file, we calculated the sample sparsity, feature variance, and mean together with differences in prevalence and the generalized fold change [6] between mock groups. Additionally, the separation between original and simulated samples in principal coordinate space was evaluated using PERMANOVA as implemented in the *vegan* package [63]. As a complementary approach, a LASSO logistic regression machine learning model was trained to discriminate between real and simulated samples using the SIAMCAT R package [10], and the AUROC of the cross-validated model was recorded. In short, real and simulated data were combined into a single feature table, with the label for classification being either “real” or “simulated” (independent of the groups used for signal implantation). After log-standardization of relative abundances, a LASSO model was trained with a ten-times repeated tenfold cross-validation scheme, and the average performance recorded.

### Included DA testing methods

To evaluate the performance of various DA testing methods, the R implementation of each method was incorporated into SIMBA using the recommended normalizations, if applicable, as described below (with default parameters if not stated otherwise, see Additional File 2: Table S2). The following methods were included in the benchmark (methods which allowed for confounder-adjustment by inclusion of covariates into the model and were assessed here are denoted with an asterisk*), usually available through an R package of the same name (version and installation routes are in the *renv.lock file* of the BAMBI repository):

*Wilcoxon: for the naive Wilcoxon test, the *wilcox.test* function available through base R was used per taxon; per default in R, the ranks of tied observations were averaged.
  - For adjusted testing, the *wilcox_test* function of the *coin* package [64] was used with formula “*feature* ~ *label | confounder”*.
- Kolmogorov–Smirnov test (*KS*): the *ks.test* function available through base R was used per taxon.
*Linear models (*LM*): for naive testing with linear models, the *lm* function available through the base R distribution was used for each taxon. *P* values were then extracted by applying the function *anova* on the trained model.
  - For adjusted testing, the confounder variable was included as a random effect in the model formula using the *lmer* function of the *lmerTest* package [53] (formula “*feature* ~ *label* + *(1|confounder)*”). This implementation produces *P* values equivalent to *MaAsLin2 * [54] with default parameters. The main grouping variable (label) was tested for significance using the base R *summary* function on the fitted *lmerModel* object. We also tested different linear model formulae, with little difference in performance (including the hypothetical equivalent of an adjusted *t*-test, i.e., a fixed effect *LM* with formula “*feature* ~ *label* + *confounder”*, see Additional File 1: Fig. S11).
- *t*-test: for the naive t-test, the *t.test* function available through base R was used per taxon.
*Limma  [65]*:* the *lmFit* function was used with the complete feature matrix and the label as design matrix as input. *P* values were then extracted after applying the *eBayes* function on the resulting *MArrayLM* object, which applies a moderated t-statistic to the linear model coefficients.
  - For adjusted testing, the confounder was supplied to the *lmFit* function call as *block* parameter.
- *edgeR * [66]: normalization factors were estimated using the *calcNormFactors* function with the *RLE* method across the whole dataset, as in Nearing et al. [67]. Then, the *estimateCommonDisp* and *estimateTagwiseDisp* functions were applied to the *DGEList* object before differential abundance testing was performed with the *exactTest* function (also in the package).
- *DESeq2 * [68]: as recommended in the *phyloseq* vignette [69], the geometric mean for each sample was added to the *DESeqDataSet* object as a normalization factor. Finally, differential abundance was calculated with the function *DESeq* which uses the *nbinomWald* test function (also in the package) as the default.
- *ALDEx2 * [51]: the *aldex* function was used with default parameters, which specify a Welch’s t-test on log-transformed and centered data.
- *mgs* and *mgs2* (*metagenomeSeq * [22]): low prevalence features (< 5% across all samples) and samples with fewer than ten counts were filtered out. As recommended in the *metagenomeSeq* vignette, a normalization factor was calculated for each sample via the *cumNormStat* function and added to the *MRExperiment* object. For testing, two different models can be fitted within the same R package, which are included here as *mgs* (using the *fitZig* function) and *mgs2* (using the *fitFeatureModel* function), analogously to Weiss et al. [25]
- *ZIBSeq * [70]: the *ZIBSeq* function in the package with the same name was used. The included option to perform a *sqrt* method-specific normalization was run separately (*ZIBSeq_sqrt*).
- *Corncob * [71]: the feature matrix was transformed into a *phyloseq* object and the *differentialTest* function was applied to test each feature (using the formula “ ~ *label*”). Per the method default and suggestion in the vignette, the Wald test was used for hypothesis testing.
- *ZINQ * [72]: the *ZINQ_tests* function was applied to each bacterial taxon, and the resulting *P* values were calculated with the *ZINQ_combination* function using default parameters.
- *distinct * [73]: the *distinct_test* function was used with default parameters.
- *ANCOM * [50]: no dedicated R package is available from the original publication and the standard implementation is prohibitively slow for larger benchmarks (see Additional File 1: Fig. S16). Therefore, we used the implementation available through Lin et al. [74]. Since *ANCOM* does not return *P* values, its primary outputs (W values) were converted into a score ranging between 0 and 1 for easier evaluation through the same framework as the other methods. The recommended decision threshold for discoveries in *ANCOM* is equal to 0.7 × number of tested taxa. Therefore, the W values above this decision threshold were transformed into scores lower than 0.05 (corresponding to a “discovery” in our evaluations), whereas all other W values were monotonically transformed to range between 0.05 and 1. Since this score does not constitute real *P* values, but rather a convenience for scoring the output of the package with the same functionality as the output of other methods, we did not apply any multiple hypothesis correction on the transformed *ANCOM* score.
- *ANCOM-BC * [47]: the *ancombc* function from the ANCOMBC package was used. The *lib_cut* parameter (indicating a minimum number of counts per sample) was set to 100. In contrast to ANCOM, ANCOM-BC outputs *P* values.
*fastANCOM  [49]: the *fastANCOM* function from the package with the same name was used with default parameters.
  - For adjusted testing, the confounder variable was passed as parameter *Z* to the *fastANCOM* function.
- *LinDA * [48]: the *linda* function in the *LinDA* package was used with the following parameters, in concordance with the GitHub README: *lib_cut* = *1000, prev.cut* = *0.1, windsor.quan* = *0.97* and raw *P*-values were extracted out of the resulting list.
- *ZicoSeq * [32]: the *ZicoSeq* function with default parameters was used (based on the *GUniFrac* package vignette), except for the following: top-end Winsorization (*winsor.end* = *'top'*), low prevalence filter (*prev.filter* = *0.1, max.abund.filter* = *0.002*), and square-root transformation (*link.func* = *list(function (x) x^0.5)*, as shown in the vignette of the package). The raw *P*-values were extracted and FDR-corrected for all subsequent analyses. *ZicoSeq* also provides a specialized permutation-based FDR control, which was not assessed in this work due to its computational burden.

### Additional preprocessing transformations

Most included DA methods take read count values as input and either work on those directly or perform specific normalizations, which are described above (and evaluated in Additional File 1: Fig. S5). To explore the effect of a preprocessing step commonly applied in microbiome data analysis, we (optionally) included *rarefaction* of counts as an additional transformation (before method-specific normalizations were applied) for all methods. This transformation consisted of downsampling counts to the 25th percentile of the total counts across samples.

The DA methods *Wilcoxon* test, *KS* test, *LM*, and *limma*, do not explicitly model count data and work with a variety of data distributions. Therefore, we applied a range of other transformations that have been widely used in the microbiome field. Overall, the applied transformations consisted of: *clr* (centered log-ratio transform), *rclr* (robust centered log-ratio transform), *TSS* (total sum scaling), *TSS.log* (total sum scaling, followed by log_10_ transformation of the data), and *TSS*.*arcsin* (total sum scaling, followed by the arcsine square root transformation), *rarefaction-TSS* (rarefaction followed by total sum scaling), and *rarefaction-TSS.log* (rarefaction followed by total sum scaling and log_10_ transformation of the data).

### Benchmarking of DA testing methods at different sample sizes

To simulate different sample sizes, we randomly selected *n* samples out of the two groups (*n*/2 from each) for each combination of effect size and each repetition. These samples were saved via indices such that for comparisons each method was applied to the exact same data. Seven different sample sizes were explored (12, 24, 50, 100, 200, 400, and 800) and 50 sets of test indices were created for each. For the evaluation of a single DA method, a total of 980,000 unique configurations were generated and used as input (7 abundance shifts × 4 prevalence shifts × 100 implantation repeats × 7 sample sizes × 50 subsamples as testing repeats).

The *P* values across all taxa were recorded and adjusted for multiple hypothesis testing using the Benjamini–Hochberg procedure [75]. If no *P* value was returned for a specific taxon (because the taxon had been filtered out by a method-specific filtering step, for example), we set this value to 1 instead before BH adjustment. To evaluate the performance of each method, we checked the raw *P* values from each testing scenario for how well bacterial taxa with differential abundance were detected, calculating an AUROC score using the raw *P* values as a predictor. The observed FDR and recall were calculated using the BH-corrected *P* values at a cutoff of 0.05.

### Generating confounded simulations through biased resampling

We identified two high-risk confounding scenarios relevant to real clinical microbiome studies [5, 6, 8, 14], representing both biological and technical factors known to influence community composition, and extended our framework in order to simulate data for benchmarking under each scenario.

For the first scenario mimicking, e.g., medication intake in a case–control disease study and impacting only few features, a confounder label was created by repeating the implantation procedure previously outlined, resulting in two distinct sets of ground truth features (each corresponding to a different random binary variable, see Fig. 3a). Alternatively, for the second scenario mimicking, e.g., technical batch effects (or study heterogeneity) in a meta-analysis, which tend to impact a majority of features, two independent real datasets were combined *before* the creation of mock case–control groups followed by implantation into only one set of ground truth features on the basis thereof, as in our earlier simulations. Here, following a single application of the implantation procedure on pre-combined input data, the confounder label was not simulated but taken from the study affiliation (Zeevi [33], TwinsUK [37], or Schirmer [38], see Additional File 1: Fig. S15). In each scenario, four distinct groups of samples were created from the combination of a mock case–control and confounder label (either simulated or from the real study affiliation).

To be able to generate confounded data for DA testing of a predefined extent (i.e., the extent of non-independence between two signals), a novel biased resampling algorithm was implemented in SIMBA and applied during the generation of testing subsets. Given a desired sample size, biased resampling is designed to calibrate the proportions of samples drawn from each of the four groups to achieve a dependency structure between the mock case–control and confounder variables. The confounder strength is encoded as the “bias” term, and is analogous to the phi coefficient as a measure of association between two binary variables. While phi is a standardized metric ranging from -1 to 1 (with 0 indicating no association and ± 1 indicating perfect positive or negative association, respectively), our confounder strengths functionally range from 0 to 1, corresponding to observations in real data where a control group is present (see Additional File 1: Fig. S10).

A bias value of 0 in our simulations represents a negative control setting, whereby all four groups are proportionally represented in each resampled testing subset; there is no association between the main and confounder variables, and hence there is no confounding (see Fig. 3a, b). Larger bias values correspond to stronger correlations between the main and confounder variables, and therefore stronger preferences for two of the groups to be present in the final testing subsets (see Fig. 3a). The induced dependency structure between the variables makes their respective ground truth signals increasingly challenging to discern from one another unless both variables are modeled (see Fig. 3b), mimicking confounding in real case–control studies.

### Effect size assessment *in real* case–control datasets

To compare simulated data to real case–control microbiome studies, we collected datasets for two diseases with a well-described microbiome signal. For colorectal cancer (CRC), we included the data from five studies [6, 76–79] conducted across three continents, which were the basis for an earlier meta-analysis that identified consistent microbial biomarkers for CRC [6]. For Crohn’s disease (CD), we similarly included five case–control studies [4, 80–83] that had been analyzed previously [10]. For CD, the data were restricted to the first measurement for each individual, whenever applicable. The data from all studies were taxonomically profiled via mOTUs2 [62] (v2.5) and features were filtered for at least 5% prevalence in at least three of the studies. Differences in prevalence across groups and the generalized fold change were calculated for each microbial feature as previously described [6] and the significance of enrichment was calculated using the blocked *Wilcoxon* test from the *coin* package in R [84].

### Confounder and robustness analysis in the MetaCardis data

To deepen our understanding of confounder effects found in real clinical microbiome data, we evaluated a subset of drug-disease combinations from the MetaCardis cohort, which were preprocessed as described above. Each disease subcohort was combined with the control group to constitute a case–control dataset, and the phi coefficient was calculated using a custom implementation of the standard formula [85] with respect to each binary drug intake metadata variable (see Additional File 1: Fig. S10a). For each bacterial taxon, two naive linear models were built using the base R *lm* function which modeled bacterial abundance as a function of either disease status or drug intake only, and two corresponding confounder-adjusted models were built using the *lmer* function from the *lmerTest* package [53], and additionally incorporated drug intake or disease status as a random effect, respectively. Significances of the resulting coefficients were adjusted for multiple testing according to the Benjamini–Hochberg procedure [75] and used to classify associations (at an FDR of 0.05).

For a given disease-drug combination, taxa which were significantly associated with the disease status and the drug intake in all four models were assigned a “drug- and disease-associated” status. Taxa bearing a significant disease association in both naive and adjusted models which did *not* possess a significant association with drug intake in the adjusted models were classified as “disease-associated”. Lastly, taxa which were significantly associated with both drug and disease in the naive models, but no longer with disease in the adjusted models, were considered to be “drug-confounded”. Taxa which had no significant associations with disease but significant drug associations in both naive and adjusted models were simply “drug-associated.”

### Implementation

The codebase for the presented results is split into two projects. The first one, an R package called SIMBA (*Si*mulation of *M*etagenomic data with *B*iological *A*ccuracy, available at https://github.com/zellerlab/SIMBA), provides the modular functionality to (i) simulate metagenomic data for a benchmarking project, (ii) perform reality checks on the simulated data, (iii) run differential abundance (DA) testing methods, and finally, (iv) evaluate the results of the tests. The second project, BAMBI (*B*enchmarking *A*nalysis of *M*icro*B*iome *I*nference methods, available at https://github.com/zellerlab/BAMBI), is a collection of R scripts relying on the *batchtools* package [86] in order to automate and parallelize the execution of SIMBA functions. Both SIMBA and BAMBI are available through GitHub and will enable other researchers to explore a similar benchmarking setting for other baseline datasets, other biomes, and additional DA testing methods. As part of the respective GitHub repositories, we included vignettes to showcase the functionality with toy examples. For reproducibility and to allow direct comparison of new methods with those in the presented benchmark, the simulation files and statistical results presented in this manuscript are available on Zenodo (see the “Availability of data and materials” section). The *renv* package manager [87] was used to install all software and to document versions, and our computing environment may be instantiated on new machines using the *renv.lock* file in the BAMBI repository.

### Data structures

To efficiently store and organize the large amount of related data required to evaluate both metagenomic simulation and differential abundance methods, we designed SIMBA around the Hierarchical Data Format (HDF5) [88]. Although we opted to work with R (v4.0.0) and the *rhdf5* package, HDF5 files are language-independent.

To ensure that the exact same input data was used for each DA test, we implemented our framework to pass a specific set of normalized feature vectors (e.g., bacterial taxon counts) to any implemented method. The samples to be included in each test are stored via their indices in the HDF5 format; for example, when testing on sample sizes *n* = 100 and *n* = 200 for 50 iterations, there would be a 50 × 100 and a 50 × 200 matrix of sample indices stored for each effect size parameterization of each simulated dataset.

## Supplementary Information

Additional File 1: Supplementary Figures.

Additional File 2: Supplementary Tables.

Additional File 3: Supplementary Notes.

Additional File 4. Review History.

## Data Availability

The synthetic datasets used in this manuscript and source data for all figures are available on Zenodo (10.5281/zenodo.8429303) [89]; this includes preprocessed species-level taxonomic profiles and metadata from all cohorts used in our analyses. For the public datasets, we downloaded the raw data from ENA using the following accessions: PRJEB11532 [90] for Zeevi WGS, ERP010708 [91] for TwinsUK WGS, and PRJNA319574 [92] for Schirmer WGS. The HMP 16S abundance tables and metadata were downloaded from the data HMP data portal at https://portal.hmpdacc.org/ [93]. The MetaCardis data are available from Zenodo (10.5281/zenodo.6242715) [94]. The software package to simulate metagenomic data (SIMBA) is available on GitHub [95] and has been submitted to the Comprehensive R Archive Network (CRAN) under the name simbaR. Similarly, the repository containing the scripts to run a benchmark or reproduce the results presented here (BAMBI) is also available on GitHub [96]. Both projects are distributed under the GPL-3 license and the version of the code described in the manuscript is available in the Zenodo repository [89] that also contains the synthetic datasets.
