# Supplementary material for: A realistic benchmark for differential abundance testing and confounder adjustment in human microbiome studies
Source: Genome Biol. 2024 Sep 25;25:247. doi: 10.1186/s13059-024-03390-9 (PMC11423519; doi:10.1186/s13059-024-03390-9)
Supplement: Supplementary file 1 — Additional File 1: Supplementary Figures. [file 13059_2024_3390_MOESM1_ESM.pdf]

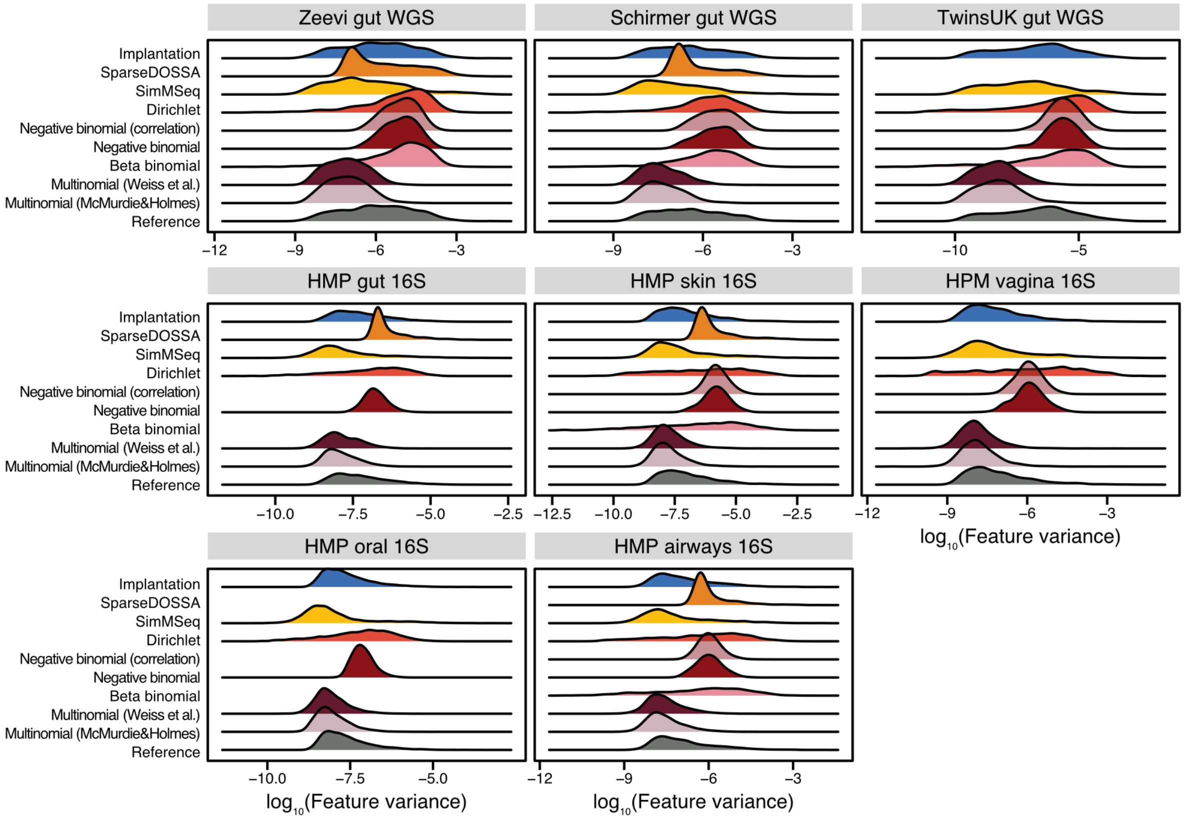

Figure S1: Distribution of feature variances is preserved in signal implantation, but not parametric simulations. For all baseline datasets (see **Methods**), the distribution of feature variances were recorded for the real input data (reference, gray) as well as simulated data from different parametric simulation frameworks or the signal implantation framework. For simulated data, the distributions are shown for a single repeat of a fixed effect size (abundance scaling of 2, prevalence shift of 0.2, if applicable). Missing ridges for some datasets indicate the simulation procedures failed to converge after five days of compute time.

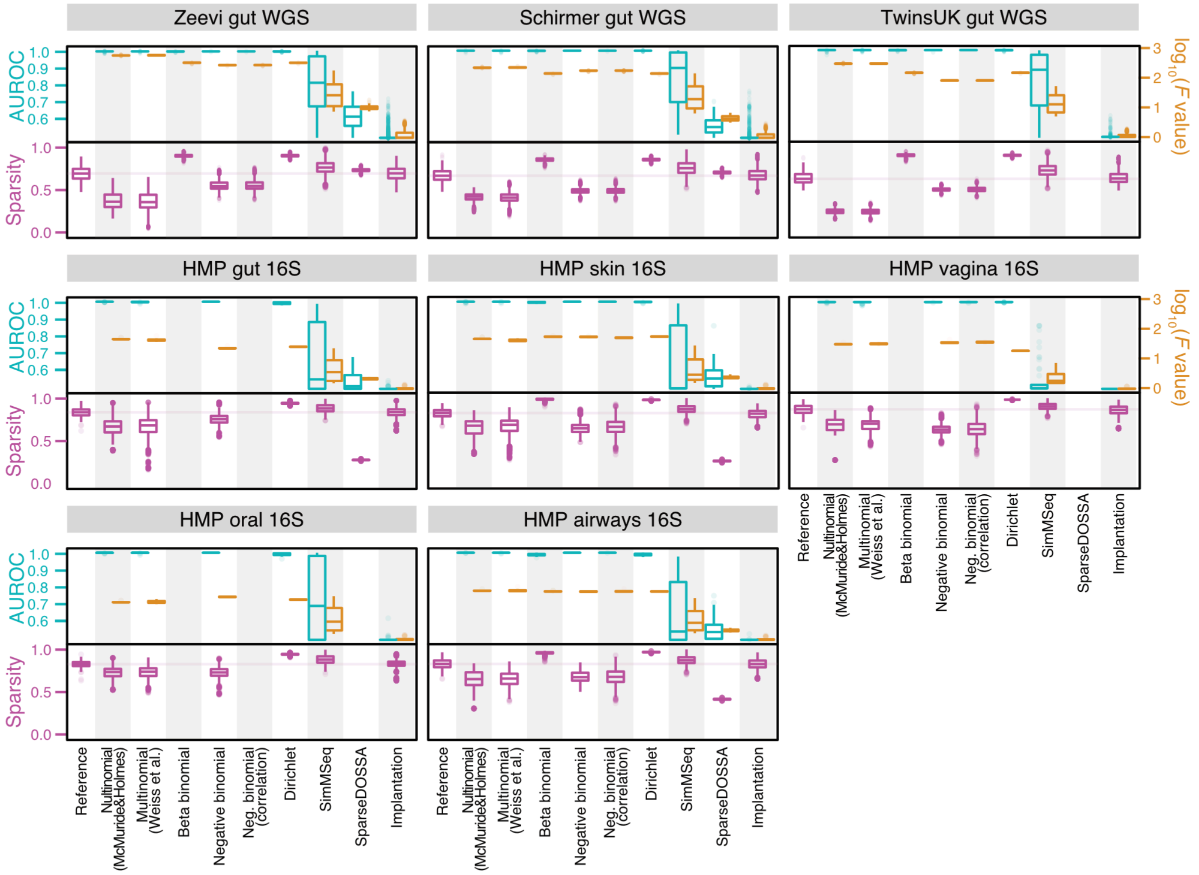

Figure S2: Sample sparsity, machine learning, and PERMANOVA analyses indicate that signal implantation, but not parametric simulations, can reproduce key data characteristics across various datasets. For all baseline datasets (see **Methods**), sample sparsity was recorded as the fraction of zero-abundance features per sample for the real input data (reference) as well as data simulated with different parametric simulation frameworks or the implantation framework. The distribution of sample sparsity is shown as magenta box plots, with the median line of the reference extended across each subplot. Similarly, the AUROC values resulting from machine learning analyses (a measure for how well a machine learning model can distinguish between real and simulated samples, see **Methods**) and the log-transformed F-values from PERMANOVA analyses (a measure for how different real and simulated samples are from one another, based on log-Euclidean distances between samples) are shown as cyan and brown boxplots, respectively. Boxplots show the measures for all repetitions (100 for the dataset from Zeevi WGS and 20 for all other datasets) and effect sizes. Missing boxes for some datasets indicate the simulation procedures failing to complete after five days of compute time. See **Fig. 1** in the main text for box definitions.

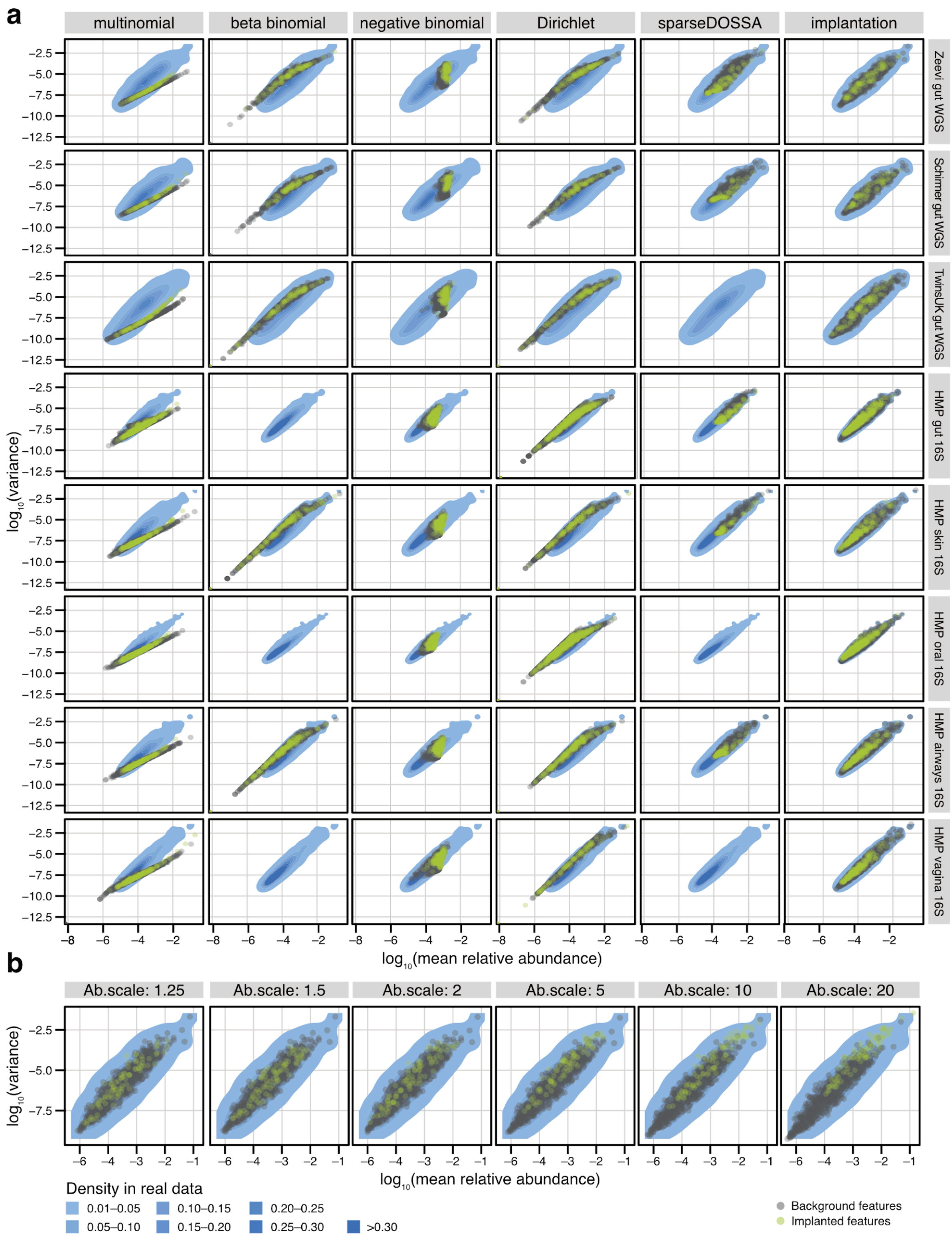

Figure S3: Mean-variance relationship is retained in data derived from signal implantation, but not parametric simulations. a) For all baseline datasets, the log10-transformed mean relative abundance is plotted against the log10-transformed variance for all taxa simulated with five parametric simulation frameworks and the signal implantation framework. A single repetition of a fixed effect size (abundance scaling of 2, prevalence scaling of 0.2, if applicable) is shown. Features that have been selected for differential abundance are highlighted in green. The blue shaded area indicates the density of the mean-variance relationship in the real input data, estimated through the `MASS::kde2d()` function in R (see color key). Missing points for some datasets indicate that the simulation procedures failed to converge after five days of compute time. **b)** For the Zeevi gut WGS dataset, the mean-variance relationship is shown for various abundance scaling effect sizes (fixed prevalence

scaling of 0.2, a single random repetition). Using the approach of the PERMDISP test (`vegan::betadisper`), the dispersion of real and simulated mean-variance values was tested for significant differences. For an abundance scaling factor of 10, 46% of repetitions resulted in significant ($P < 0.05$) differences in the dispersion between real and simulation data, depending on the implanted prevalence shift (prevalence shift of 0: 7%, pr. shift of 0.1: 40%, pr. shift of 0.2: 60%, and pr. shift of 0.3: 77% of repetitions), whereas 97% of repetitions for abundance scaling of 20 showed significant differences. None of the tests for other abundance scaling effect sizes were significant. All parametric simulation frameworks resulted in data with significantly different dispersion between real and simulated mean-variance values irrespective of the effect size used.

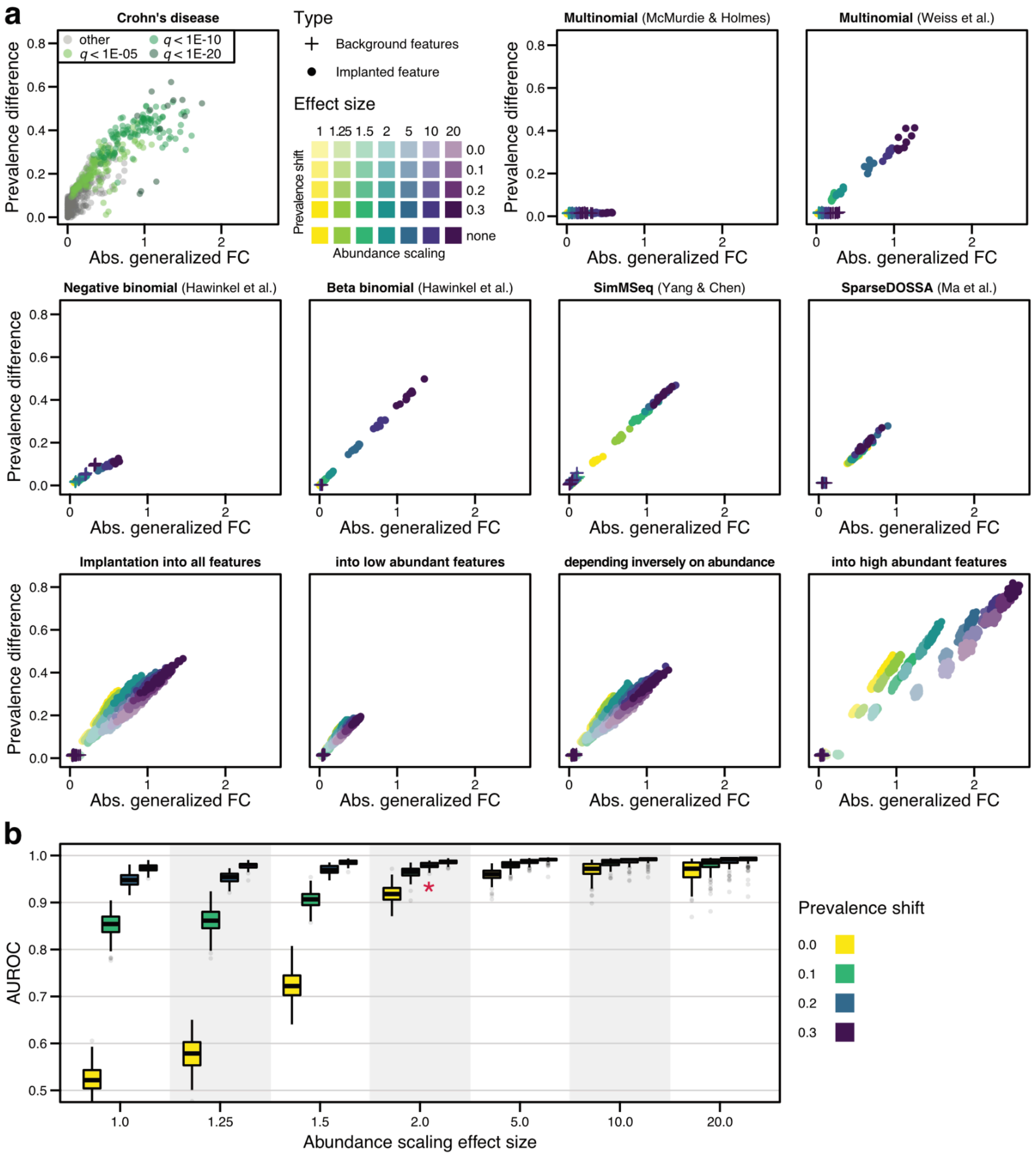

Figure S4: Implanted effect sizes vary across different simulation schemes and eligible feature sets. a) The absolute generalized fold change (gFC)[6] and the absolute prevalence difference between groups was calculated for all features across all repetitions in every simulation scheme. For each repetition, the mean gFC and mean prevalence difference values were calculated for both background and implanted features (ground

truth differentially abundant features). As a reference, the real gFC and prevalence shift values observed across all features in the Crohn's disease meta-analysis are shown in the top left panel (see **Methods** and main text **Fig. 1**, also for a comparison to gFCs and prevalence shifts observed in colorectal cancer). In the bottom row, mean gFC and mean prevalence shift values are shown for different signal implantation configurations that vary feature sets eligible for implantation (see **Methods**). Signals implanted into high abundant features resulted in (mean) effect sizes which were much larger and deviated substantially from real Crohn's disease data. **b)** Boxplots show the AUROC values for distinguishing between implanted and background features, using the gFC values as predictor. All values were calculated for the simulation based on the Zeevi WGS dataset, with all features eligible for implantation. Except for small effect sizes, the gFC values are almost perfectly separated between background and implanted features, indicating that the implanted features have higher gFC values than background features. The effect size shown in the main figures is highlighted by a red star.

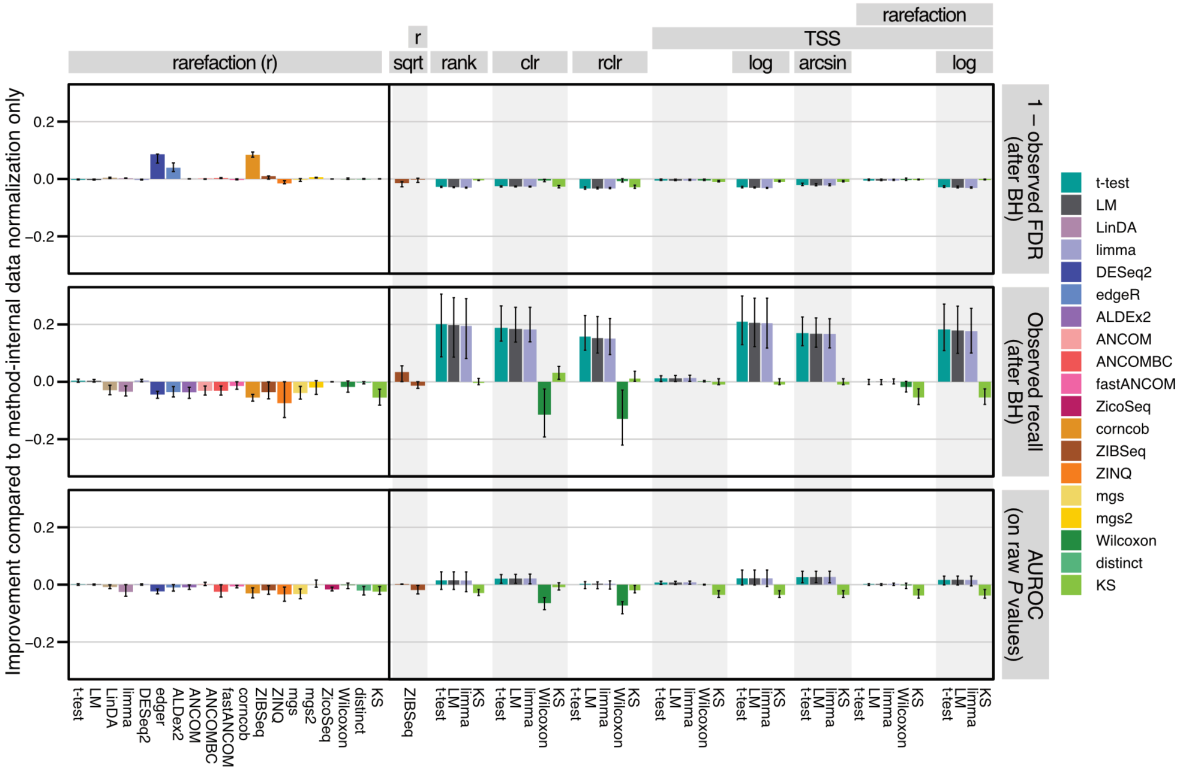

Figure S5: Performance of differential abundance testing methods tends to decrease after rarefaction. The mean change in observed FDR, observed recall and AUROC for the detection of ground truth differentially abundant (DA) features are shown as bars across all tested effect and sample sizes to compare method-internal normalization (exclusively) with prior data transformation (assessing methods that are commonly used in microbiome data analysis, such as e.g. rarefaction). Negative values denote decreased performance when compared to no data preprocessing, and error bars indicate the standard deviation across all repetitions. The horizontal bars on the top of the plot indicate the preprocessing method used (and combinations thereof, e.g. total sum scaling (TSS) could be combined with rarefaction and/or other downstream transformations). For all DA testing methods, rarefaction was applied before any method-specific normalization was performed. Overall, rarefaction led to a decrease in observed recall and lower AUROC scores across most methods. Other data preprocessing methods were applied for methods that do not specifically model count data and could therefore be supplied with other types of data. Both *limma* and the *LM* showed marked improvements in recall (with comparably low deterioration of observed FDR) across several preprocessing techniques, where the compositional preprocessing methods *clr* and *rclr* lowered the AUROC and the recall for detection of ground truth DA features for the *Wilcoxon* test.

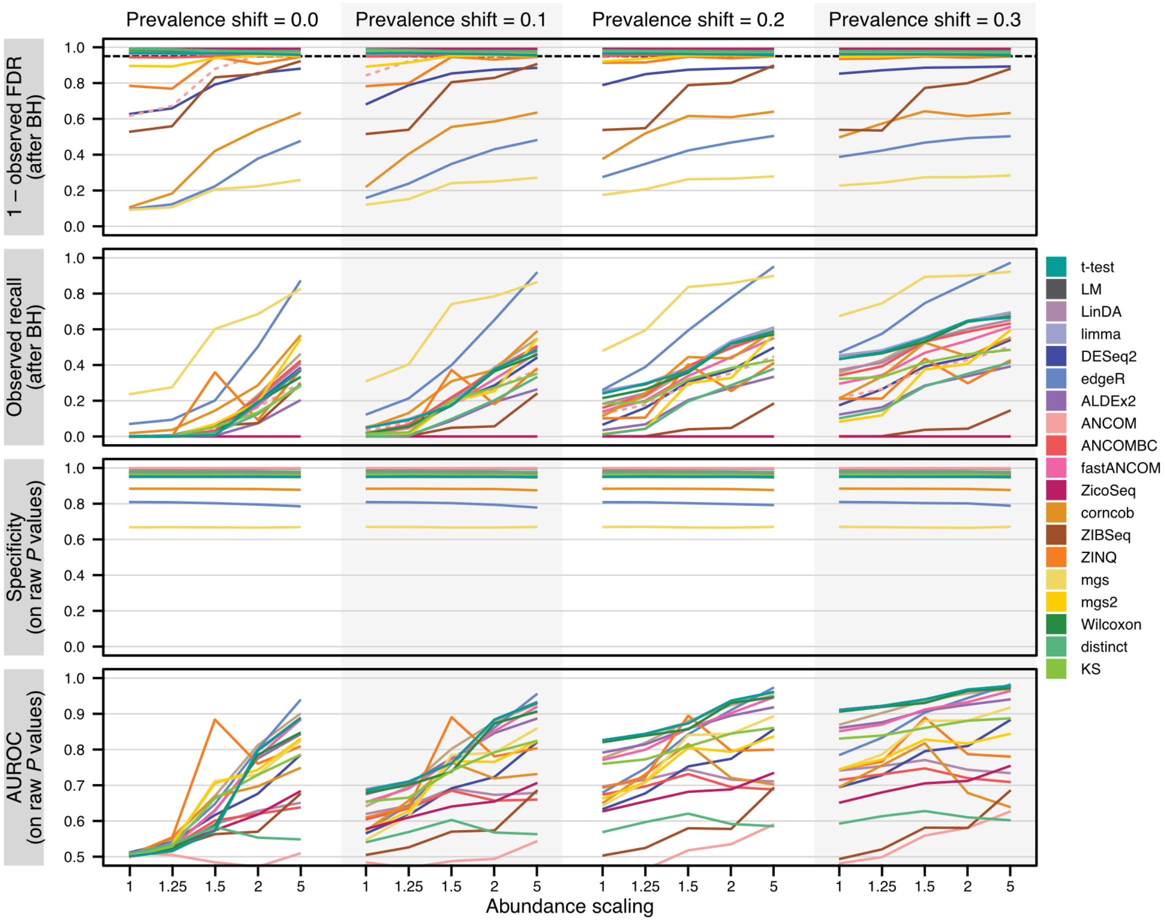

Figure S6: Performance of differential abundance testing methods improves with increasing effect size. The mean observed FDR, mean observed recall, the mean specificity (1 - false positive rate, calculated on raw *P* values) and the mean AUROC (on raw *P* values) for the detection of ground truth DA features are shown across all included methods for varying effect sizes of the same signal implantation benchmark (all features eligible for implantation). All values were recorded for a sample size of 100. With larger effect sizes (both prevalence shifts and scaled abundances), the AUROC for the detection of ground truth DA features generally increased. For ANCOM, lines for mean observed FDR and recall are dashed, since ANCOM does not output *P* values and multiple testing corrections cannot be applied (see **Methods**).

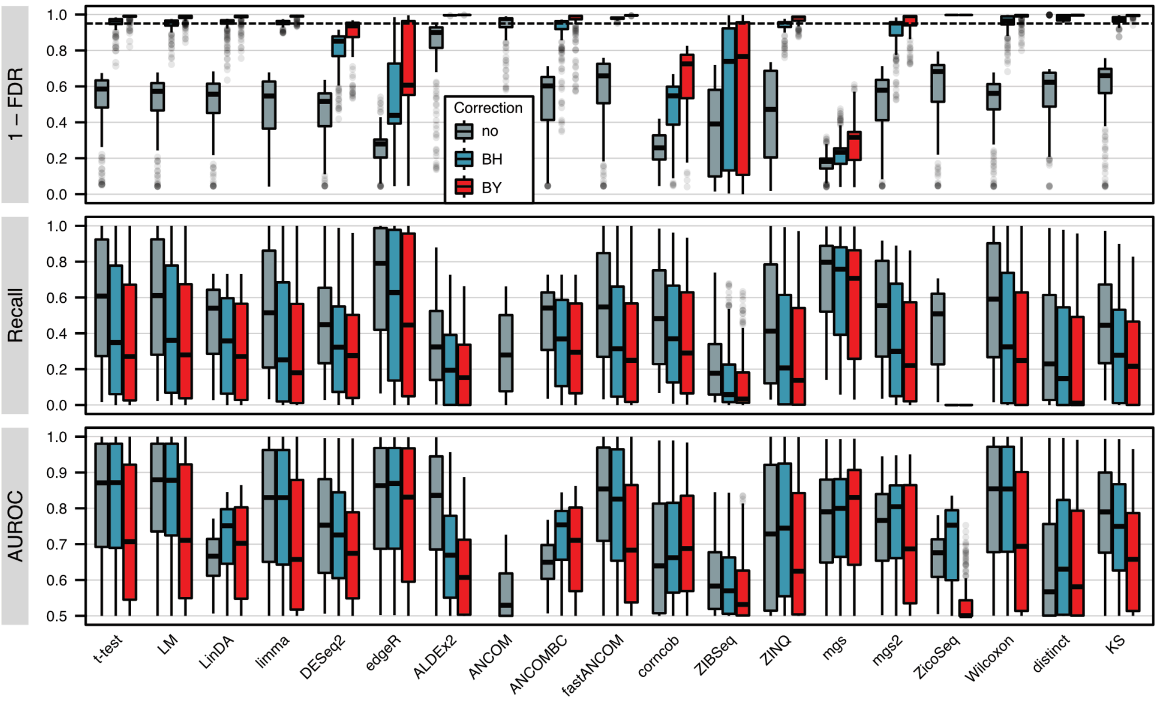

Figure S7: The effect of different adjustment methods for multiple hypothesis testing. For each included DA testing method, FDR, recall, and AUROC for the detection of differentially abundant features was calculated at a cutoff of 5%, using either the raw *P* values (no correction), or the results of the Benjamini-Hochberg correction (BH), or the Benjamini-Yekutieli correction (BY). Box plots show the distribution of all values across all repetitions and included effect sizes. As expected, a cutoff of 5% on raw *P* values results in high FDR across all methods except for *ALDEx2*, which appears to be overconservative – in line with previous benchmarks[45]. After either BH and BY correction, most methods properly control the observed FDR at the nominal 5% level, while those methods that failed to do so under BH, also failed under BY (e.g. *metagenomeSeq (mgs)*, *edgeR*, or *corncob*). The BY correction not only results in lower observed FDR across all methods, but it also generally leads to reduced recall and lower AUROC. See **Fig. 1** in the main text for boxplot definitions. Since ANCOM does not output *P* values (see **Methods**), BH and BY corrections cannot be applied, so the corresponding values cannot be shown.

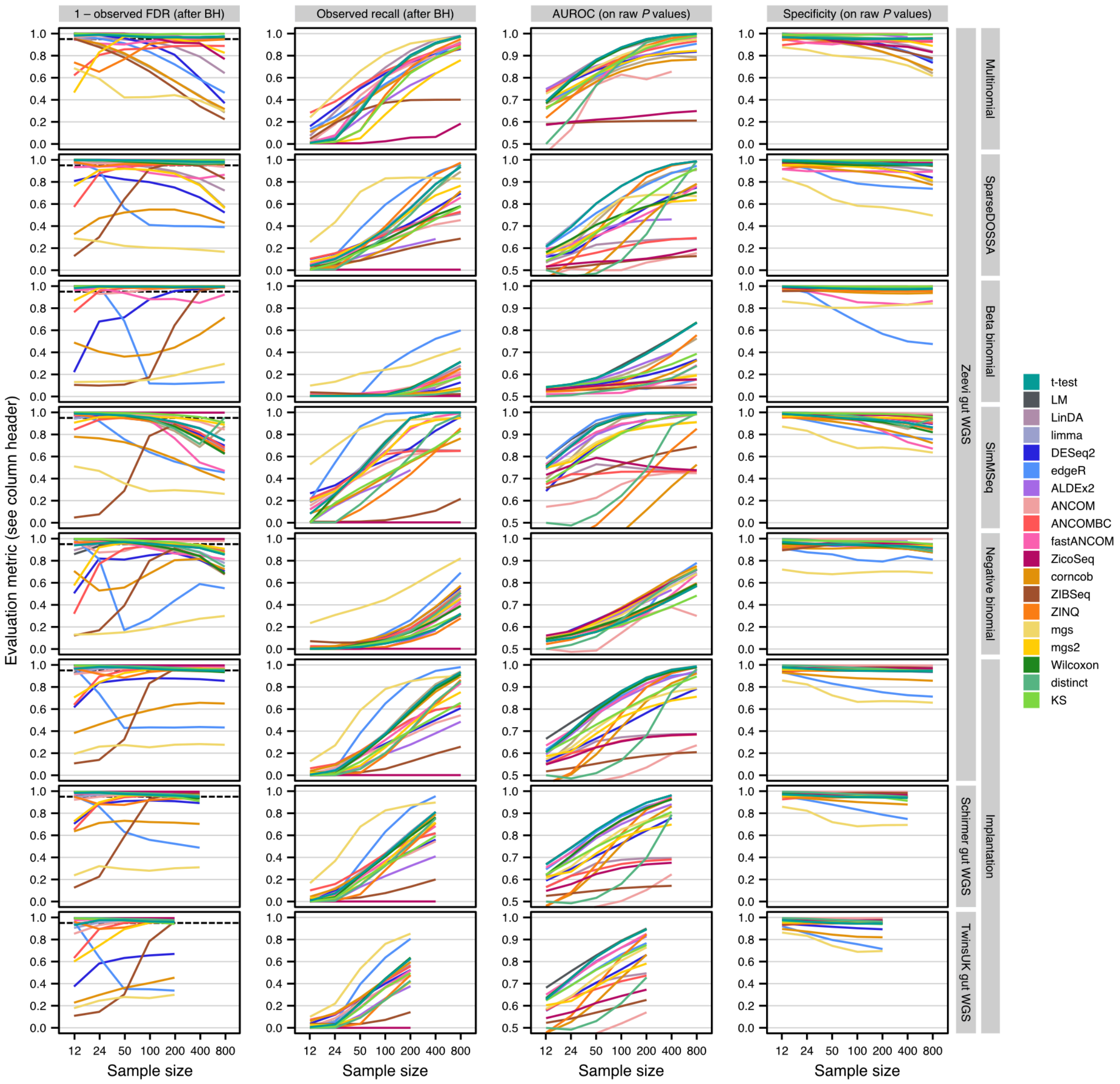

Figure S8: Performance of differential abundance testing methods varies largely across simulation frameworks and comparably little across datasets. The evaluation results of different underlying simulation frameworks are visually compared across the top 6 rows (see labels to the right) and contrasted to the differences in evaluations resulting from the use of different data sets in the implantation framework across the bottom three rows. For a single, moderate effect size (abundance scaling of 2, prevalence shift of 0.1, if applicable see **Fig. 2**), the mean observed FDR, mean observed recall (both computed after BH correction of raw *P* values), the mean specificity, and mean AUROC values for the detection of differentially abundant features are shown across all repetitions for all included DA testing methods (see also **Methods**). Data simulated by parametric simulation frameworks used the real Zeevi gut WGS dataset as input. For ANCOM, lines for observed FDR and recall are dashed, since ANCOM does not output *P* values (see **Methods**).

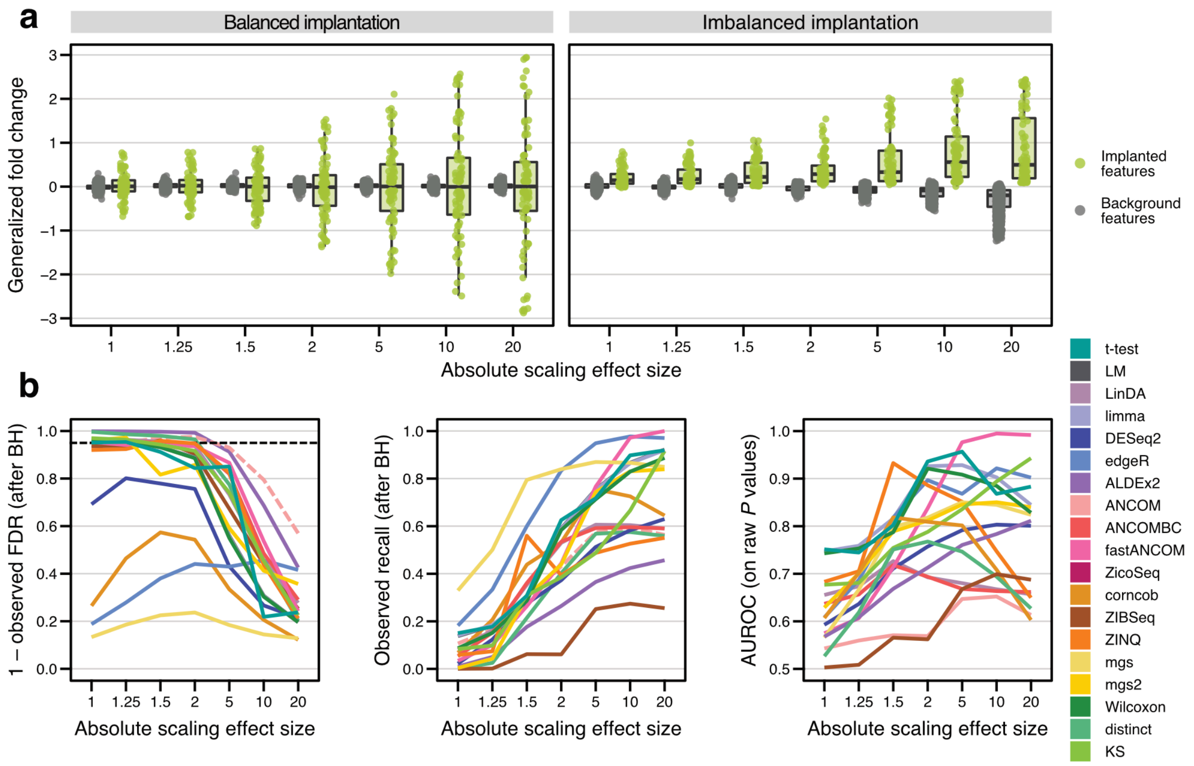

Figure S9: Performance of differential abundance testing methods in the presence of strong compositionality effects. a) The generalized fold change is shown across different effect sizes for background features and implanted features for the default signal implantation framework with a balanced implantation into both groups (left, see **Methods** and Weiss *et al.*[25]) and the imbalanced implantation (right, see **Methods** and also Jonsson *et al.*[30]). In the default framework, compositional effects are minimized, meaning that background (unimplanted) features show no difference between groups, whereas in the imbalanced implantations, features are only implanted into a single group, thereby leading to abundance shifts in background features (average generalized fold change decreasing with effect size) as a consequence of compositionality. Generalized fold changes for a single representative repetition are shown (prevalence shift of 0.2, only high abundant features eligible for implantation, see **Fig. 1** in the main text for boxplot definition). **b)** The mean observed FDR, mean observed recall, and mean AUROC values are shown across all repetitions (prevalence shift of 0.2, only high abundant features eligible for implantation, sample size of 200) for each abundance scaling effect size (with which the compositionality effects also increase) for all included DA methods (see **Methods**). While compositionality (at extreme effect sizes) leads to a general trend of deteriorating DA method performance, *ANCOM* and *ALDEx2* are least affected in terms of FDR. For ANCOM, lines for observed FDR and recall are dashed, since ANCOM does not output *P* values (see **Methods**).

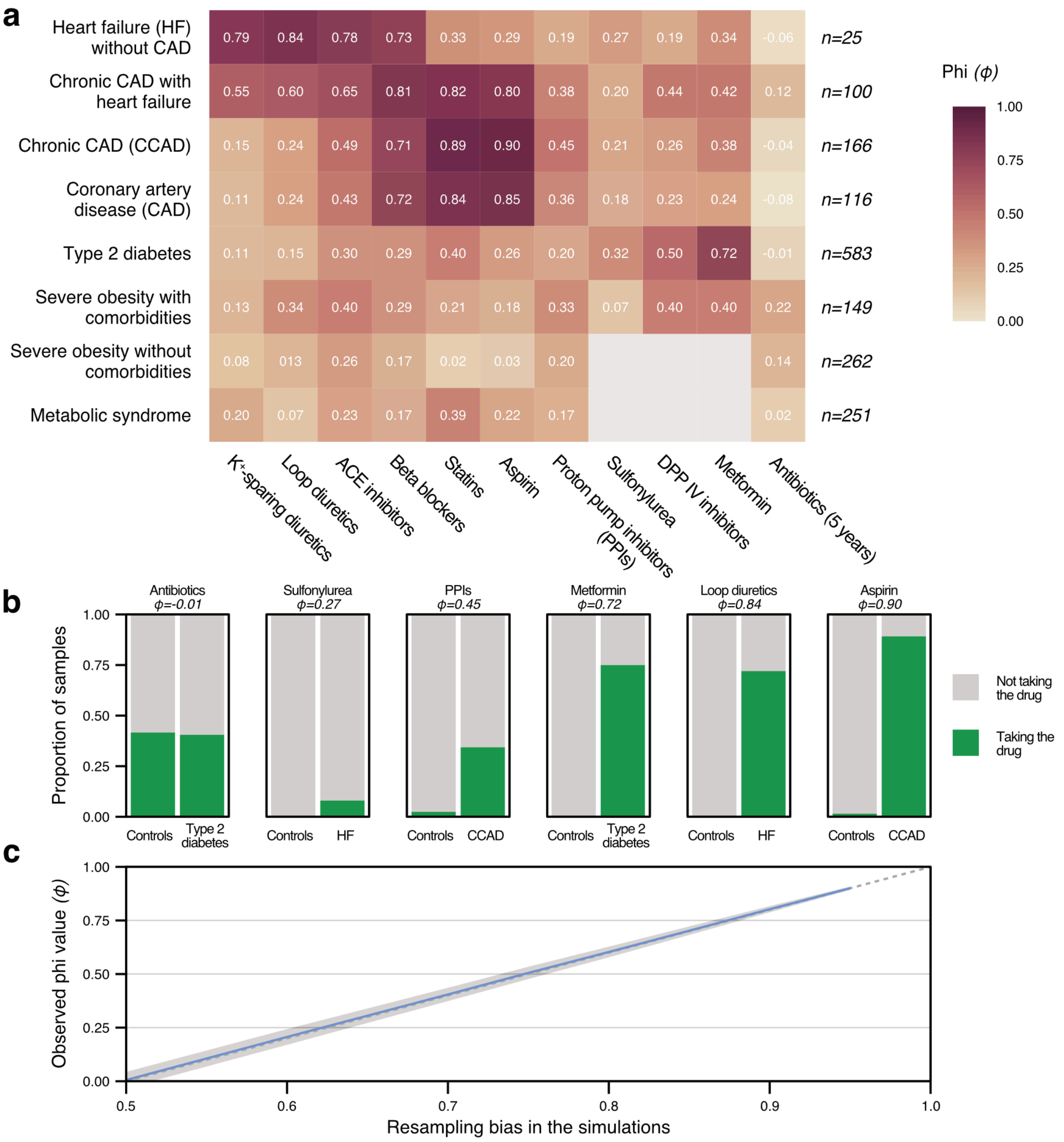

Figure S10: Empirical phi coefficients between cardiometabolic diseases and medication. a) Phi coefficients were calculated between different disease groups from the MetaCardis cohort[14] and n=330 healthy controls (see **Methods**). For a given disease, the highest phi values are observed for the most common drug indications. Gray squares represent NAs, which resulted when no individuals in either case or control group were taking a given medication. **b)** Medication intake broken down by case or control group. High concomitance between drug intake and disease status produces large phi coefficients, while negative coefficients indicate that the control group was more medicated than a given disease cohort. **c)** Linear relationship (with 95% confidence interval as shaded gray area) between the bias parameter used in our framework to produce confounded simulations (see **Methods**) and empirical phi values of simulated data.

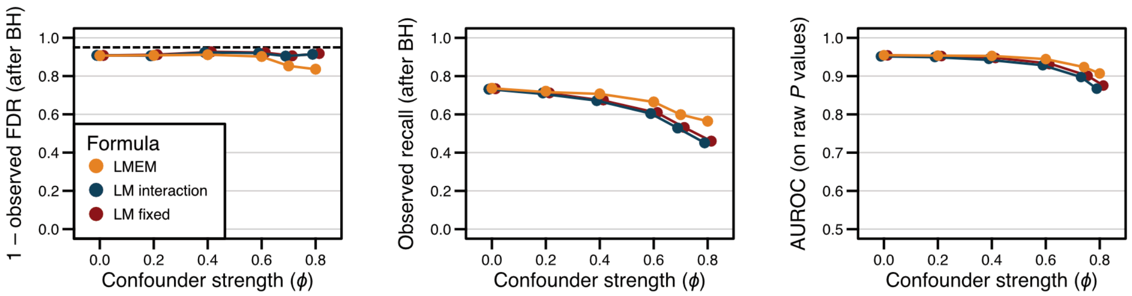

Figure S11: Performance of confounder-adjusted linear models is minimally impacted by choice of model formula. Different ways of adjusting the linear model for confounders were explored for the same simulation as shown as in **Fig. 3** (abundance scaling factor of 2, prevalence shift of 0.2, all features eligible for implantation, sample size of 200 shown here). LMEM represents the random effect model used in the main text for confounder adjustment, i.e. with formula *lmerTest::lmer(feature~label + (1|confounder)),* which was fit using the base R *summary* function. The LM interaction model had the formula *lm(feature~label * confounder)* and was also fit with the *summary* function. The fixed effect model was implemented as *lm(feature~label + confounder),* and the significance of the label variable was tested using a Type III analysis of variance (via the *car::Anova* function), i.e. one which does not depend on the order of terms in the model formula. The LMEM had a slightly higher proportion of false positives than the other methods at confounder strengths > 0.6, albeit coupled with higher recall and AUROC, but results were largely similar to one another in our evaluation.

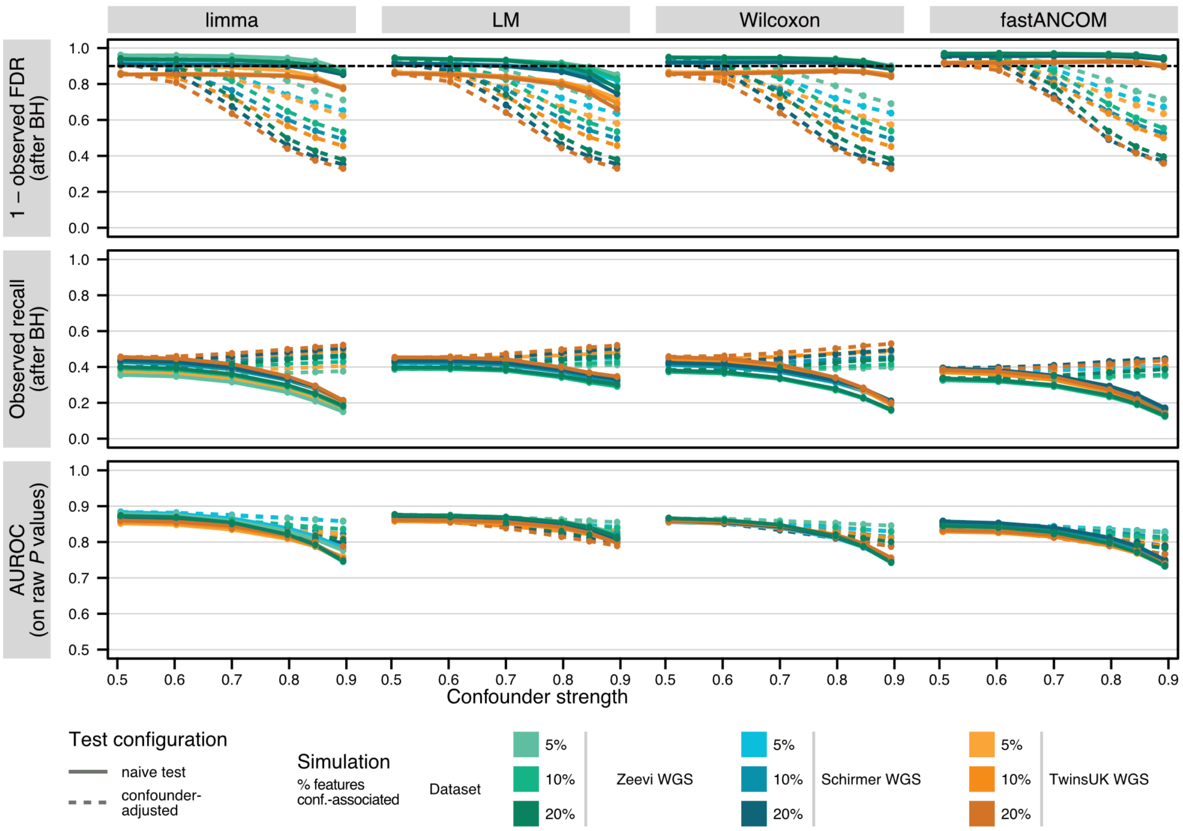

Figure S12: Confounder-adjusted DA testing methods show similar performance across datasets and varying proportion of confounder-associated features. For all included gut WGS studies (Zeevi WGS, Schirmer WGS, and TwinsUK WGS), several confounded simulations were created with a varying number of features implanted as confounder-associated features into the simulations (5%, 10% or 20% of features implanted with the confounder label, always 10% of features implanted with the main group label, see **Methods**). The mean observed FDR, the mean observed recall, and the AUROC values for the detection of differentially abundant features were calculated for each simulation and each of the included DA testing methods (*limma*, the *LM*, the *Wilcoxon* test, and *fastANCOM*), while taking either the confounder variable into account or not (naive and confounder-adjusted models, respectively). Lines show the mean performance of each method across all repetitions for a single effect size (abundance scaling of 2, prevalence shift of 0.2) for each different simulation. All values were recorded for a sample size of 100.

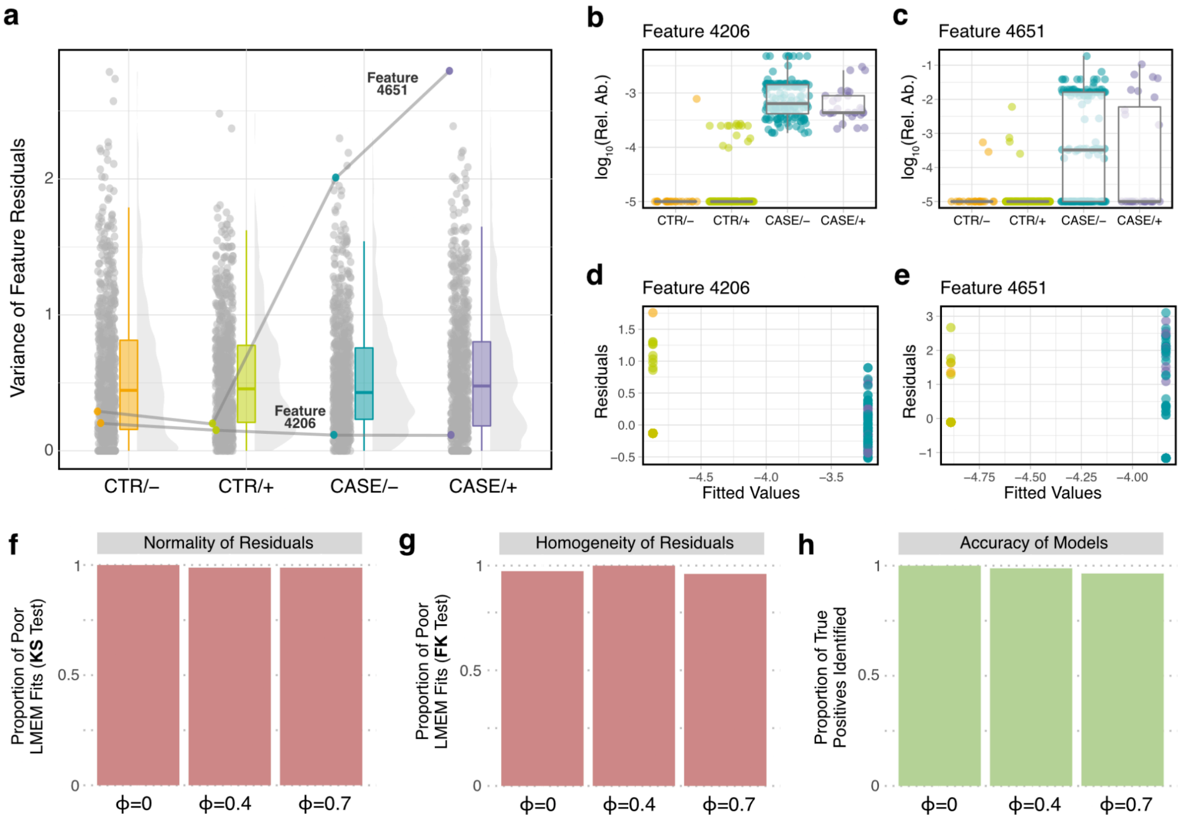

Figure S13: Linear mixed-effect models are able to distinguish the ground truth with high accuracy, despite poor adherence to model assumptions. a) Using the sample size 200 from the same representative confounded simulation from the main text **Fig. 3,** residual variances of mixed-effect models were extracted from all bacterial taxa for each simulated group (CASE/CTR reflects the main group label, whereas +/- indicates the confounder label, see **Methods**). Identified features represent models of ground truth taxa with the smallest and largest residual variances between groups, indicating the best and worst adherence to model assumptions (Features 4206 and 4651, respectively). **b-c)** Log-transformed relative abundances which were used as input for the LM and LMEM in our benchmarks for the features in a. **d-e)** Fitted values and residuals for the features in a, showing clear separation of the case-control groups by their fitted means in both cases. **f-g)** For each ground truth feature (N=84 total), fifty models were built for each confounder strength (quantified by ϕ, reflecting the same confounder strengths in main text **Fig. 3b**), corresponding to the same random testing subsets used for benchmarking (see **Methods**), and their fits were quantified via non-parametric statistical tests. While visual inspection of residuals is advised, it does not scale well to the high-throughput nature of DA testing or benchmarking. The Kolmogorov-Smirnov (KS) test was used to compare residuals against a standard normal reference distribution, and the Fligner-Killeen (FK) test evaluates the homogeneity of variances across groups. *P* values were adjusted for multiple testing using the Benjamini-Hochberg method, then averaged across the 50 repetitions. Models with adjusted *P* values ≤ 0.05 violated assumptions of residual normality and/or homogeneity of group variances respectively, and were considered poor fits. Regardless of confounder strength, more than 95% of ground truth features did *not* meet LMEM assumptions. **h)** Coefficients for the main group (case-control) label were extracted from the exact same models as in f and g, adjusted for multiple testing, and classified as either true positives or false negatives using the same adjusted cutoff (≤ 0.05 for true positives). Echoing our findings in the main text (evaluated across many more simulations and repetitions), LMEMs were slightly less accurate under high confounding (ϕ=0.7).

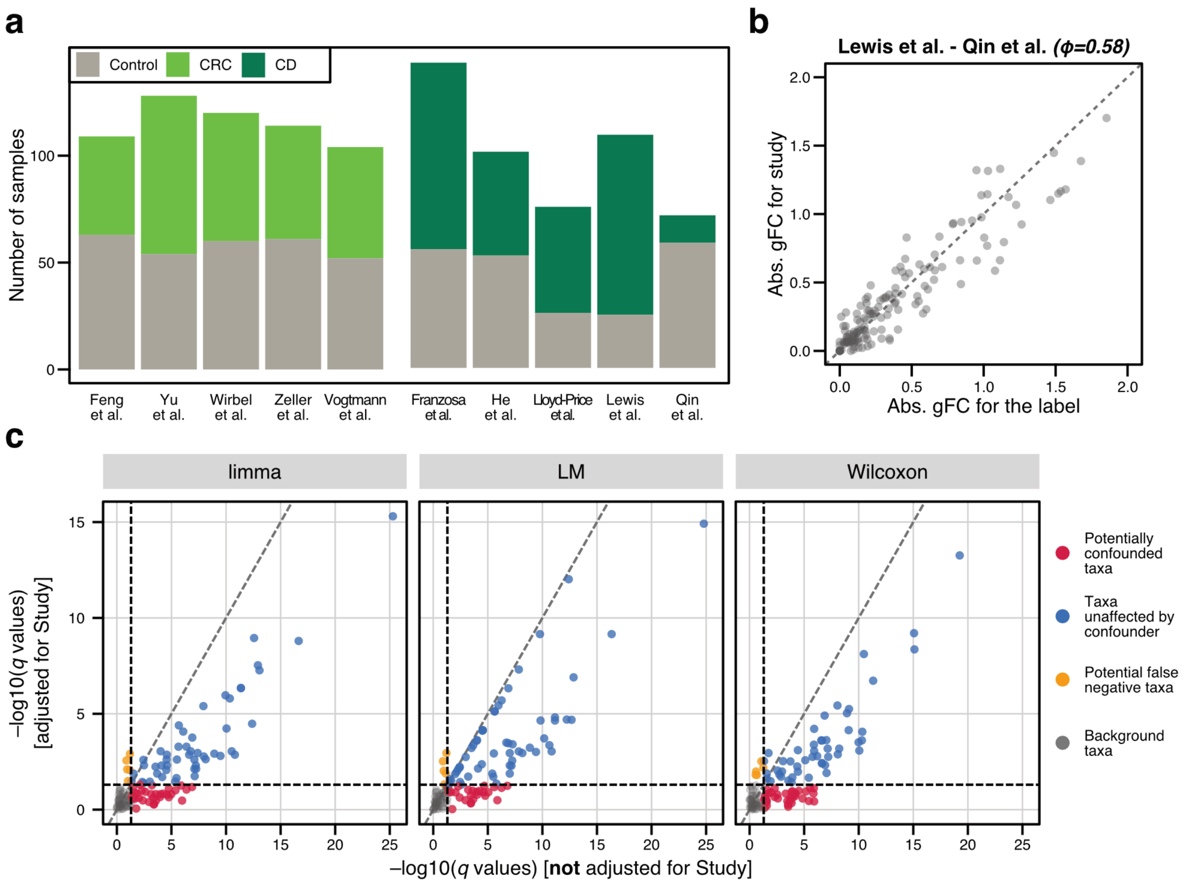

Figure S14: Study heterogeneity between real datasets resembles confounding conditions achieved using signal implantation and biased resampling. a) For both colorectal cancer (CRC) and Crohn's disease (CD), the number of samples in each group (control and respective case group) is shown across studies as a bar plot. For CRC, studies are generally balanced, while there are larger differences in group proportions for CD studies. **b)** Across all pairwise combinations of CD studies, Lewis *et al.*[81] and Qin *et al.*[80] exhibit the strongest confounding potential due to study heterogeneity (ϕ=0.58 between study origin and disease status). For this study combination, the generalized fold change associated with the disease label (CD) and the study origin are plotted against each other across all included bacterial taxa, showing a pronounced correlation. **c)** Using both the naive (not adjusted for study heterogeneity as a confounder) and the study-adjusted configuration of *limma*, the *LM*, and the *Wilcoxon* test, all bacterial genera from the combination of Lewis *et al.* and Qin *et al.* (see b) were tested for differential abundance between control and CD samples. The resulting estimated FDR values (Benjamini-Hochberg corrected *P* values) are plotted against each other as scatter plots, with 0.05 indicated as dashed black line for both models. Red dots highlight taxa that are potentially confounded (identified as differentially abundant in the naive model, but not the confounder-adjusted model), with a similar proportion of potentially confounded taxa across all three DA testing methods. Taxa that are significantly associated with CD, independent of the type of model used, are highlighted in blue, whereas taxa that were not significant in the naive model, but do appear significantly different in the confounder-adjusted model are shown in orange.

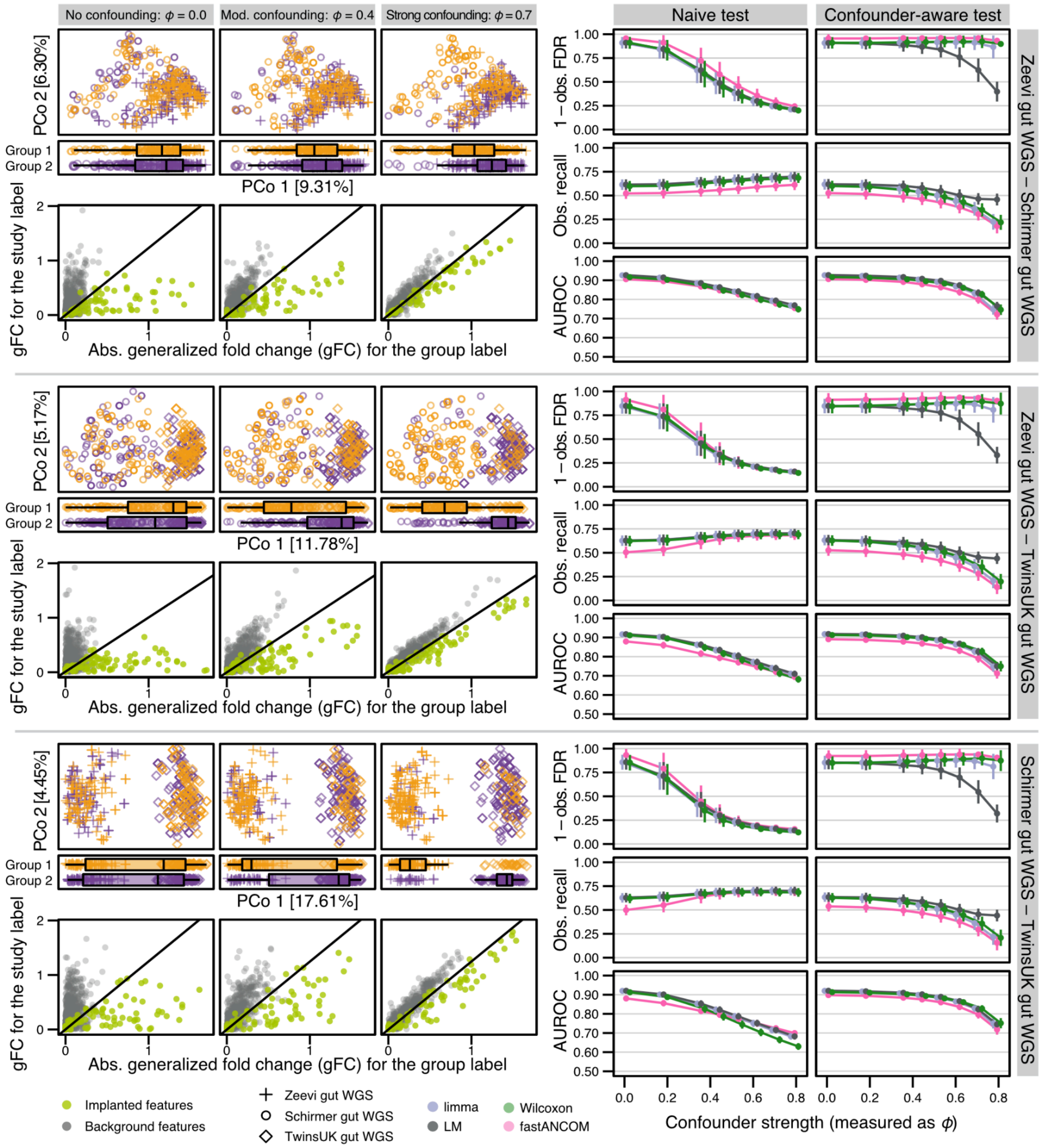

Figure S15: Broad confounding results in slightly worse performance of DA testing methods compared to narrow confounding. For each two-way combination of the included gut WGS studies (Zeevi WGS, Schirmer WGS, and TwinsUK WGS), implantation simulations were created from data of both studies as input, using the study information as confounding variable for the generation of (biased) resampled testing sets (see **Methods**). On the left, principle coordinate projections for various levels of confounding are shown, visualizing how the study and group variables become more aligned with increased confounding strength (as seen in the group-resolved boxplots for the first principle coordinate). Underneath, the absolute generalized fold change (gFC) for the group label is contrasted with the gFC for the study label, with implanted features highlighted in light green (see **Fig. 3** in the main text for reference). On the right side, the performance of *limma*, the *LM*, *fastANCOM,* and the *Wilcoxon* test are shown in dependence of the confounder strength as measured by ϕ (see **Methods**). Each test was run in the 'naive' (without adjusting for the study variable, left column) and in the confounder-adjusted configuration (right column). Observed (abbreviated to obs.) FDR and recall were calculated after Benjamini-Hochberg correction, while AUROC was calculated on raw *P* values.

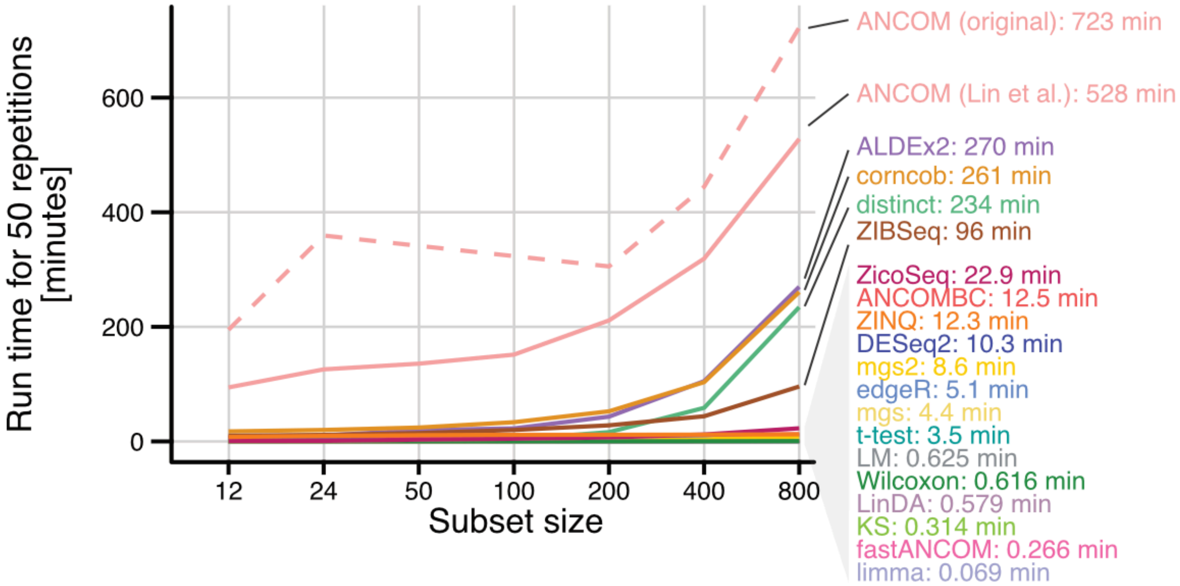

Figure S16: Comparison of runtime across differential abundance testing methods. Runtime was recorded on the same machine for 50 repetition of different subset sizes from a single repetition in the same signal implantation benchmark (abundance scaling of 2, prevalence shift of 0.1, all features eligible for implantation). Methods are annotated with the time needed to run the subset size of 800 samples on the same laptop. The original *ANCOM* implementation was obtained from the website of the first author of the *ANCOM* manuscript at https://sites.google.com/site/siddharthamandal1985/research.
