# Supplementary material for: A realistic benchmark for differential abundance testing and confounder adjustment in human microbiome studies
Source: Genome Biol. 2024 Sep 25;25:247. doi: 10.1186/s13059-024-03390-9 (PMC11423519; doi:10.1186/s13059-024-03390-9)
Supplement: Supplementary file 2 — Additional File 2: Supplementary Tables. [file 13059_2024_3390_MOESM2_ESM.pdf]

Table S1: Overview of DA Method Benchmarks

Benchmark	*Simulations Employed*	*Samples per Group*	*Type of Count Data*	*No. DA Methods*	*DA Method Co-Release*
Paulson *et al.*, 2013[22]	Custom parametric (log-normal sample counts + logistic ZI/sparsity model)	Not reported	16S	7	metagenome Seq[22]
**McMurdie and Holmes, 2014**[23]	**Multinomial** from GlobalPatterns	N=(3, 5, 10)	16S	5	–
Johnsson *et al.*, 2016[30]	Non-parametric downsampling approach. No code available.	N=(3, 6, 10)	WGS (genes only)	5	–
Thorsen *et al.*, 2016[34]	Multiplicative and/or additive spike-in and rescale. No code available.	N=(16, 50)	16S	10-12	–
**Weiss *et al.*, 2017**[25]	**Multinomial**, Dirmult, Gamma-Poisson from GlobalPatterns	N=(5, 40)	16S	6-8	–
**Hawinkel *et al.*, 2019**[26]	**Negative Binomial, Beta Binomial,** SimSeq (non-parametric)	N=(5, 25, 100)	16S, WGS (taxa)	10	–
Calgaro *et al.*, 2020[27]	Negative Binomial and ZINB for microbiome data	N=(10, 20, 40)	16S, WGS (taxa), (sc)RNA	11	–
**Mallick *et al.*, 2021**[54]	**SparseDOSSA**[31]	N=(5, 10, 25, 50, 100)	WGS (taxa, genes)	15-18	MaAsLin2[54]
Nearing *et al.*, 2022	None	–	38 real datasets	14	–
Capellato *et al.*, 2022[89]	metaSPARsim[90] (semi-parametric MHG)	N=(10, 25, 50, 100)	16S	9-12	–
**Yang and Chen, 2022**[32]	**SimMSeq**, (semi-parametric Bayesian, CoDA)	N=(25, 50, 100)	16S, WGS (taxa)	13-18	ZicoSeq[32]
Kodalci *et al.*, 2023[52]	SPsimSeq[91] (semi-parametric, developed for (sc)RNA-seq)	N=(25, 50, 75)	16S	8	Signtrans[52]
Wirbel *et al.* (this study)	SIMBA (signal implantation + bolded simulations above)	N=(6, 12, 24, 50, 100, 200, 400)	16S, WGS (taxa, genes)	19	–

Key study metadata from differential abundance (DA) benchmarks applied to microbiome data from the last decade. Bolded studies were at least partially reproduced as part of this study. Simulation frameworks and/or strategies, samples per group, type(s) of count data explored, and number (no.) of DA methods tested are detailed. A range of DA methods is listed since some benchmarks gave special emphasis to method-normalization combinations. If a benchmark was performed as a (supporting) part of a manuscript introducing a new DA method, the method is listed (and the benchmark should not be considered neutral[92]). See **Additional File 3: Supplementary Note 1** for a longer discussion about the impact of restricted sample size and data types on benchmark conclusions.

ZI, zero-inflated; **16S**, 16S rRNA gene; **WGS**, whole (meta-)genome sequencing; **(sc)RNA**, (single cell) RNA-seq; **ZINB**, zero-inflated negative binomial; **MHG**, multivariate hypergeometric; **CoDA**, compositional differential abundance.

Table S2 : Differential abundance methods implemented in this study

DA Method	*Type*	*Norm.*	*Trans.*	*Assumed count distribution*	*Hypothesis test*	*R pkg.*
***limma**[65]	1	TSS	Log	–	Moderated t-test	3.46.0
**DESeq2**[68]	1	Geometric mean	–	Negative binomial	Wald test	1.30.0
**edgeR**[66]	1	TMM	–	Negative binomial	Exact test	3.32.1
**distinct**[73]	1	–	–	–	NP CDF permutation	1.2.0
**ALDEx2**[51]	1, 2	–	CLR	Dirichlet-multinomial	Wilcoxon rank sum	1.22.0
**mgs**[22] (*fitZig*)	2	CSS	Log	ZI Gaussian (MM)	Moderated t-test	1.32.0
**mgs2**[22] (*fitFeatureModel)*	2	CSS	Log	ZI Log normal (MM)	Moderated t-test	1.32.0
**ANCOM**[50]	2	Indirect	ALR	–	Custom, based on log-ratios	–
**ANCOM-BC**[47]	2	Bias correction	Log	–	Custom, based on Wald test	1.0.2
***fastANCOM**[49]	2	–	Log	–	Custom, based on Wald test	0.0.4
**ZicoSeq**[32]	2	Ref. taxa selection	Win. + Sqrt.	–	Smith permutation	1.8
**LinDA**[48]	2	Bias correction	Win. + CLR	–	Custom, based on Wald test	1.1
**ZINQ**[72]	2	–	–	–	Custom, based on ZI and quantiles	1.0
**ZIBSeq**[70]	2	TSS	Sqrt.	ZI Beta binomial	Wald test	1.2
**corncob**[71]	2	None	–	Beta binomial	Wald test	0.1.0
**KS**	3	–	–	–	KS test	Base R
**T-test**	3	TSS	Log	–	Student's t-test	Base R
***Wilcoxon**	3	–	–	–	Wilcoxon rank sum	Base R
**Blocked Wilcoxon**[64]	3	–	–	–	Permuted Wilcoxon rank sum	*coin* 1.4-1
***LM** (*lm)*	3	TSS	Log	–	ANOVA F test	Base R
**LMEM**[53] (*lmer)*	3	TSS	Log	–	Approx. F test	*lmerTest* 3.1-3

Key attributes and implemented settings of all 19 benchmarked methods, where Type reflects the data for which it was designed (Type 1=RNA-seq, Type 2=microbiome, and Type 3=all/classical statistics). Asterisks (*) denote which methods were included in the stratified DA testing benchmark as well. For fastANCOM and limma the same packages were used for both benchmarks, but the blocked Wilcoxon and LMEM were used to extend the Wilcoxon and LM for stratified DA testing (from the coin and lmerTest packages, respectively). Source package names (if relevant) are listed in the last column if they differ from the method name; similarly, function names are identified in parentheses next to the method name. Normalization and transformation recorded here reflect published recommendations, if available, otherwise they reflect settings that yielded the best performance (see **Fig. S5**). The space in this table is not sufficient to explain the complex pre-processing and/or hypothesis testing procedures of several; for example, representatives of the ANCOM tool family all make use of a W score and

DA Method	*Type*	*Norm.*	*Trans.*	*Assumed count distribution*	*Hypothesis test*	*R pkg.*

additional decision-making. The impact of rarefying counts as a (sequence depth) normalization technique was also explored for all methods, see **Fig. S5**.

Norm., normalization; **Trans.**, transformation; **Pkg.**, package; **NP**, non-parametric; **CDF**, cumulative density function; **MM**, mixture model; **KS**, Kolmogorov-Smirnov test; **LM**, linear model; **LMEM**, linear mixed-effect model; **mgs**, metagenomeSeq; **RLE**, relative log expression; **ZI**, zero-inflated; **TMM**, Trimmed Mean of M-values; **TSS**, total sum scaling; **Win.**, Winsorization; **Sqrt.**, square root transformation; **CLR**, centered log ratio; **ALR**, additive log ratio.
