# Supplementary material for: A realistic benchmark for differential abundance testing and confounder adjustment in human microbiome studies
Source: Genome Biol. 2024 Sep 25;25:247. doi: 10.1186/s13059-024-03390-9 (PMC11423519; doi:10.1186/s13059-024-03390-9)
Supplement: Supplementary file 3 — Additional File 3: Supplementary Notes. [file 13059_2024_3390_MOESM3_ESM.pdf]

Note S1: On the need to make fewer assumptions in large-scale, heterogeneous studies

Our work was aimed at closing the gap between existing benchmark criteria and the contemporary reality of microbiome data analysis. In designing our simulations, we considered the fundamental task of differential abundance testing of case-control taxonomic profiles. We chose this specific application because it is at the core of most human disease association studies (including those based on 16S rRNA gene markers), and because bacterial taxa are known to vary substantially between individuals (whereas the functional properties exhibited by those taxa collectively are relatively stable, see e.g. Figure 2 from the Human Microbiome Project Consortium[1]). Furthermore, taxonomic profiles in particular have demonstrably different statistical properties than simulated datasets thus far used in DA method benchmarks[22,23,25,30] (see main text **Fig. 1a**), making their recommendations potentially unreliable for real microbiome association studies. A previous benchmark by Hawinkel *et al.*[26] showed that some DA methods report spuriously low *P* values under the null hypothesis being true (especially in sparse taxa with low abundances), suggesting both misapplication and/or method-inherent insufficient type I error control in taxonomic settings.

To better characterize this behavior and estimate its impact in applied microbiome studies, we used simulations which we demonstrated to retain key characteristics of several real input datasets (including multiple body sites), and we included additional scenarios (i.e. confounding) and DA methods in our benchmark. Our findings were found to agree with those in Hawinkel *et al.* yet also greatly expanded the scope of inquiry, allowing us to more empirically delineate conditions affecting method performance.

Bulk RNA-seq methods were originally developed for few biological replicates (in contrast to the scope and scale of current microbiome research[13,93]), and test the null hypothesis that features have the same mean under two conditions (which is not robust to outliers or high between-sample variance characteristic of taxonomic microbiome profiles). To test their suitability in a large-N setting, Li *et al.*[94] use several real datasets from the Genotype-Tissue Expression (GTEx[95]) and the Cancer Genome Atlas (TCGA[96]) and show that tools like *DESeq2* and *edgeR* display exaggerated false positives in population-level RNA-seq studies (containing fifty to hundreds of samples per group). These authors also find the *Wilcoxon* test's more conservative, rank-based hypothesis to empirically outperform other methods, especially in terms of FDR control (verified by permutation). They conclude that parametric methods should only be used when the per-condition sample size is less than eight and power is a concern.

Our related conclusion that restrictive parametric methods do not offer advantages over classical statistical methods when applied to human-associated bacterial profiles – and even have negative consequences – contradicts the results from previous microbiome-specific benchmarks. These discrepancies can be explained by differences in the design of the underlying simulation strategies. Jonsson *et al.*[30] concluded *DESeq2* and *edgeR* to have the best overall performance – probably owing to their small sample sizes (two groups of 3, 6 and 10 vs. two groups of 6, 12, 25, 50 100, 200, 400, and 800 here) and exclusive use of gene-level abundances, which are well-established[1] to display far lower between-sample variance than the sparse taxonomic profiles investigated here. Reaching a similar conclusion, McMurdie and Holmes[23] explored two groups of 3, 5, and 10, and used parametric multinomial simulations, which insufficiently capture relevant statistical properties of taxonomic profiles (see main text **Fig. 1a-c**). As we demonstrate, their conclusions are not

supported when larger sample sizes and more realistic simulation procedures are considered (see **Additional File 1: Fig. S8**).

Note S2: Theoretical considerations for calibrating different methods

In our benchmark we discovered a high observed false positive rate for several methods independent of the multiple hypothesis correction procedure used (Benjamini-Hochberg or Benjamini-Yekutieli, see **Additional File 1: Fig. S7**), suggesting method-inherent issues with type I error control. One obvious culprit for this is of course unmet distributional assumptions embedded in the DA models, but another possible explanation for these high observed FDR values could be that some methods are not well-calibrated for microbiome data and report universally low *P* values, while still being able to give lower *P* values to implanted features compared to background features – in essence, correctly ranking ground truth bacterial taxa. Such cases would result in high AUROC values on uncorrected *P* values, and performance could theoretically be improved by changing the *P* value cutoff for significance. However, of those methods with high observed FDR, only *edgeR* showed comparably high AUROC values (see main text **Fig. 2**) and therefore fit this pattern, whereas other methods (such as *metagenomeSeq* or *corncob*) could not even theoretically be recalibrated to high precision and high recall.
