# Supplementary material for: A realistic benchmark for differential abundance testing and confounder adjustment in human microbiome studies
Source: Genome Biol. 2024 Sep 25;25:247. doi: 10.1186/s13059-024-03390-9 (PMC11423519; doi:10.1186/s13059-024-03390-9)
Supplement: Supplementary file 4 — Additional File 4. Review History. [file 13059_2024_3390_MOESM4_ESM.docx]

**1^st^ round**

**Reviewer 1**

The authors describe a project evaluating the performance of various methods for differential abundance (DA) analysis in microbiome studies. While this step of analysis is extremely important for any group interested in studying natural populations of microbes, there has not yet been (to my knowledge) an independent evaluation effort such has been performed for other parts of the analysis process (such as CAMI for taxonomic classification or binning).

My understanding of the literature is that most evaluation efforts are conducted by groups which are primarily concerned with presenting a novel DA approach, which may bias their design of an evaluation framework.

The key innovation presented by the authors is an in silico spike-in approach by which the abundance of specific taxa are manipulated from real-world microbiome datasets, which provides a strong basis to evaluate the sensitivity of signal detection.

Point 1.
The motivation for the implantation approach described by the authors seems well founded, but I was left with a lingering question about the methodological details for this approach. Reading over the methods on pages marked 17-18, I was left with the impression that signal implantation was performed in only one of randomly selected groups. In other words, it seemed like abundances were scaled up by a known factor for 10% of features in a random 50% of samples. Then, "the number of counts for each sample was scaled down to the original value of the unaltered sample by rarefaction". Assuming that my understanding is correct, my concern is that if abundances are scaled up and then back down then the magnitude of the implanted signal is not as large as originally intended. Moreover, the features which were not selected for scaling up would effectively be scaled down as a side-effect (which could have unintended side-effects for FDR estimation).

Of course, the authors may be able to address this by describing the degree of rarefaction that was required, which may be low depending on which features were selected. If my understanding of the text is incorrect and some factors were scaled up while others were scaled down (as would seem more obvious to me), then it might be good to update the text to make this evident. I would appreciate any clarification on this point which can be offered.

Point 2.
Figure 2 a-c is not formatted in a way that can be easily comprehended due to the large number of colors that are used (let alone the accommodation of colorblind people). One suggestion would be to add a marginal annotation pointing the method label for each line. This figure needs to be updated in some way if the reader is to gain any sort of understanding from it.

Point 3.
Figure 2d seems incomplete -- I do not understand why values are not shown for all methods and why values have been censored for some methods and some simulations. Rather than presenting incomplete data it seems like the authors should simply present the complete table and pair it with the FDR metrics which are currently being used as the basis for filtering.

Point 4.
I'm curious why corncob was not included in the confounder analysis? I only bring it up because corncob is the method with which I am most familiar (full disclosure, I have published papers with the corncob author) and it seems easily compatible with confounders (by adding the confounder as a term in the formula)

**Reviewer 2**

Wirbel et al have reviewed several prior simulation / benchmarking studies on WGS and 16S DA methods in a comprehensive report, "A realistic benchmark for the identification of differentially abundant taxa in (confounded) human microbiome studies". This reviewer believes the claimed uniqueness to their study is in the claim that 'previous DA benchmarks relying on simulated datasets did not quantitatively evaluate the similarity to real data, which undermines their recommendations'. Leveraging that premise, they shift, scale, sample etc. from existing datasets to and then evaluate common methods. There are several major concerns that I believe can be addressed; however, previous reports have already performed simulations on 'existing' datasets perturbed or randomly sampled with shifts leading to similar results.

Major concerns:
1. Quite a few benchmarking studies that have been performed in the past. This includes leveraging very similar approaches to that of Wirbel et al. The novelty in assessing the PCo coordinates of 'real', 'simulation / perturbation', etc. is unique. However, the reviewer has a difficulty in reviewing prior benchmarking studies assessed and is aware of one in particular that has randomly sampled existing study samples and assigned novel case status and scaled abundances.
a. Some of the more simple benchmarking studies have occurred as far back as 2016 where samples underwent the same process as described in Wirbel et al https://link.springer.com/article/10.1186/s40168-016-0208-8#Fig1
b. I am not suggesting a multi-'true' cohort (think n-dimensional comparison with multiple underlying studies, ie. Not just the zeevi et al study), but I would include at a minimum a table of all prior benchmarking studies and their approaches.
2. Missing methods:
a. While Wirbel et al have cited the biorxiv paper of Mallick H et al (MaAslin2) and used several methods implemented within the R package. The paper has however, was accepted in 2021 and has over 700 google citations. This reviewer would appreciate the inclusion of the primary Maaslin2 method for comparison.
i. Personally, I believe the standard t-test to be robust, but this method is catching the microbiome community by storm and is relevant enough to compare to
3. The authors do not provide enough evidence in the discussion surrounding their claims that, 'Based on our results, we argue that the failure to produce consensus is partially explained by their reliance on unvalidated and ultimately unrealistic parametric simulations of microbiome data as ground truth.'
a. Authors have not performed an exhaustive analysis of similar disease cohorts and assessed the heterogeneity in raw abundances. There exist many meta-analyses, e.g. https://www.nature.com/articles/s41467-017-01973-8 that have adequately come to conclusions of relevant organisms implicated in disease
4. The inclusion of the methods scripts is nice, but for true broad generalizability and impact / use the authors should create a sustainable Bioconductor (at a minimum CRAN) R package like sparsedossa or <https://www.ncbi.nlm.nih.gov/pmc/articles/PMC7047923/>

**Reviewer 3**

This is a well wri+en and interes/ng manuscript. It contains two important contribu/ons: (1)

a new simula/on framework for simula/ng realis/c microbiome data, and (2) a benchmark

of many DA methods. Moreover, it also evaluates methods in the presence of confounding

effects.

Comments:

1. There is one more microbiome simula/on method that I am aware of, and which also

succeeds well in simula/ng realis/c data. SPSimSeq (Assefa et al., 2020, 2023) was

originally developed for simula/ng (bulk) RNASeq data, but it has been demonstrated

to work also well for microbiome data (16S rRNA sequencing); see Kodalci and Thas

(2023).

2. For the performance evalua/on you compare the empirical FDR with the es#mated

FDR. What is the es#mated FDR?

3. Lin and Peddada (2020) argue that microbiome data oVen shows a differen/al

sampling frac/on between the two groups. Their method (ANCOM-BS), which is also

included in your study, is supposed to adjust for it. Does your simula/on method

allow for the introduc/on of differen/al sampling frac/ons? A related issue: the

library size distribu/on may be different between the two groups. If this happens,

then it is either by coincidence, or because of technical issues. Anyway, it may affect

the DA results. Your simula/on method starts with randomly spli]ng the set of all

samples into two groups, and hence, on average, no difference between the

sampling distribu/ons is expected. Would it be possible to introduce this flexibility in

the R implementa/on? I am not necessarily sugges/ng that you need to extend your

simula/on study in this way, but it could be added to the discussion sec/on.

4. In the Methods sec/on you give a clear descrip/on of the simula/on method. It is

men/oned that aVer scaling and shiVing, the generalised fold change is computed

for all taxa, and if a fold change for a an implanted feature is smaller than 0.001, then

that feature is removed. This makes sense to me, but what about the other way

around? Suppose that aVer randomly spli]ng samples into two groups, a non-DA

feature shows – by coincidence – a large fold change?

5. Whereas the descrip/on of the simula/on method in the absence of confounders is

very clear to me, this does not apply to the descrip/on of the genera/on of

confounding effects (p. 21). Although I think I can more or less what you did, I am

afraid that the descrip/on is insufficiently clear for most people to reproduce the

simula/ons, and reproducibility is important. Can you please try to provide a be+er

descrip/on?

6. With the linear models, I wonder why you have chosen to adjust for confounders by

adding them as random effects to the model? Don’t most people add the confounder

as a fixed effect to the model? For the Wilcoxon rank sum test, you have chosen for

stra/fica/on if I am correct. This is more like considering the confounder as a fixed

effect. By the way, note that Kodalci and Thas (2023) proposed extensions of the

Wilcoxon rank sum test that allows for the adjustment of con/nuous

confounders/covariates.

7. When using linear models, the confounders are added as addi/ve, linear effects.

Does this agree with the way the data is simulated? For the real data analyses, you

could e.g. make residual plots to check this assump/on.

8. Throughout the manuscript you use the term “post hoc” adjustment. I have doubts

about this terminology. Post hoc seems to suggest that the analysis is done in a

second-stage, aVer having seen the results of the first-stage data analysis. For

example, in a classical ANOVA, mul/ple comparisons of means is some/mes referred

to as post hoc tes/ng, because in the first stage the ANOVA F-test indicated an effect

of the factor variable.

References

Assefa, A. T., Vandesompele, J., & Thas, O. (2020). SPsimSeq: semi-parametric simula/on of

bulk and single-cell RNA-sequencing data. Bioinforma#cs, 36(10), 3276-3278.

Assefa A, Hawinkel S, Vandesompele J, Thas O, R Core Team (2023). SPsimSeq: Semi-

parametric simula#on tool for bulk and single-cell RNA sequencing data. R package version

1.12.0.

Kodalci, L., & Thas, O. (2023). Simple and flexible sign and rank-based methods for tes/ng for

differen/al abundance in microbiome studies. Plos one, 18(9), e0292055.

Lin, H., & Peddada, S. D. (2020). Analysis of composi/ons of microbiomes with bias

correc/on. Nature communica#ons, 11(1), 3514.

**Authors’ response**

Reviewer 1

The authors describe a project evaluating the performance of various methods for differential

abundance (DA) analysis in microbiome studies. While this step of analysis is extremely

important for any group interested in studying natural populations of microbes, there has not

yet been (to my knowledge) an independent evaluation effort such has been performed for

other parts of the analysis process (such as CAMI for taxonomic classification or binning).

My understanding of the literature is that most evaluation efforts are conducted by groups

which are primarily concerned with presenting a novel DA approach, which may bias their

design of an evaluation framework.

The key innovation presented by the authors is an in silico spike-in approach by which the

abundance of specific taxa are manipulated from real-world microbiome datasets, which

provides a strong basis to evaluate the sensitivity of signal detection.

We would like to thank the reviewer for their positive feedback on our manuscript and their

valuable and constructive comments.

Point 1.

The motivation for the implantation approach described by the authors seems well founded,

but I was left with a lingering question about the methodological details for this approach.

Reading over the methods on pages marked 17-18, I was left with the impression that

signal implantation was performed in only one of randomly selected groups. In other

words, it seemed like abundances were scaled up by a known factor for 10% of features in a

random 50% of samples. Then, "the number of counts for each sample was scaled down to

the original value of the unaltered sample by rarefaction". Assuming that my understanding

is correct, my concern is that if abundances are scaled up and then back down then the

magnitude of the implanted signal is not as large as originally intended. Moreover, the

features which were not selected for scaling up would effectively be scaled down as a side-

effect (which could have unintended side-effects for FDR estimation).

Of course, the authors may be able to address this by describing the degree of rarefaction

that was required, which may be low depending on which features were selected. If my

understanding of the text is incorrect and some factors were scaled up while others were

scaled down (as would seem more obvious to me), then it might be good to update the text

to make this evident. I would appreciate any clarification on this point which can be offered.

Response:

We appreciate the reviewer’s attention to detail here and hope we can clarify. Per default in

its implementation, our method alternates between the randomly selected groups when

implanting signals in order to mitigate the precise side-effect the reviewer mentions

(described on page 8, lines 294-296). This was inspired by Weiss et al., who made a similar

adjustment to the simulation strategy of McMurdie and Holmes to rectify the issue the

reviewer is concerned with.

The text that the reviewer cites refers to a non-default implementation that we carried out to

characterise the side-effect in question (see SFig. 9), namely confounding by compositional

effects. We found that this imbalanced introduction of signals in this particular simulation of

ours (signal implantation into only one group) led to all methods unilaterally producing more

false discoveries, exactly as expected by the reviewer (and ourselves).

Actions taken:

We adjusted the wording in the Methods section to better clarify the distinction between the

different implantation strategies (page 17, lines 638-641).

Point 2.

Figure 2 a-c is not formatted in a way that can be easily comprehended due to the large

number of colors that are used (let alone the accommodation of colorblind people). One

suggestion would be to add a marginal annotation pointing the method label for each line.

This figure needs to be updated in some way if the reader is to gain any sort of

understanding from it.

Response:

We appreciate the reviewer’s feedback and agree that the figure panels were difficult to

parse without annotations.

Actions taken:

As suggested, we added marginal annotations to Figure 2a-c, focusing on poorly performing

methods in panels a) and b). We additionally changed the figure legend for Figure 2 to

reflect those changes (pages 7-8).

Point 3.

Figure 2d seems incomplete -- I do not understand why values are not shown for all methods

and why values have been censored for some methods and some simulations. Rather than

presenting incomplete data it seems like the authors should simply present the complete

table and pair it with the FDR metrics which are currently being used as the basis for

filtering.

Response:

We thank the reviewer for this comment and agree that the previous figure seemed to

present incomplete data. For our ranking, we had not included those methods that exhibited

elevated FDR values based on a relatively lenient filtering criterion and we welcome the

opportunity to clarify that methods with poor FDR control were not considered for ranking

and subsequent analyses (see response to the next point).

During the revision, we noticed that some values were left empty because the mean AUROC

for the method was smaller than 0.5 (the colour gradient ranges from 0.5 to 1) and in two

cases, the ANCOM method could not produce results due to its extreme runtime

requirements. For each combination of effect size, sample size, and repetition, we allotted

48 hours of runtime per method, which was sufficient for all methods to produce results –

except for ANCOM on two simulations. We indicated those cases in the revised figure.

Actions taken:

We adjusted Figure 2d by adding the AUROC values also for those methods that were

filtered out for ranking based on a high FDR, indicating the distinction between these

methods and all others by using a grey-scale colour-scheme instead of the green colour

gradient used for methods not violating the FDR thresholds (pages 7-8).

Point 4.

I'm curious why corncob was not included in the confounder analysis? I only bring it up

because corncob is the method with which I am most familiar (full disclosure, I have

published papers with the corncob author) and it seems easily compatible with confounders

(by adding the confounder as a term in the formula).

Response:

We thank the reviewer for the opportunity to clarify our approach for the confounded

simulations. As the reviewer stated correctly, corncob’s implementation allows for a

confounder to be added to the model formula. Indeed, several of the models included in our

non-confounded (“naive”) benchmark can be adjusted for confounders, but were not

included in our confounded benchmarking based on their performance in the “naive”

benchmark. We reasoned that a method that produces excess false positive discoveries in

the non-confounded condition would not be able to produce satisfactory results given the

additional challenge of confounding factors.

We appreciate the reviewer’s transparency and therefore implemented corncob for the

confounded simulations as well. The confounder variable was added as an additional term in

the model formula and we ran corncob on a single iteration of the confounded simulation

based on the Zeevi WGS dataset, abundance scaling of 2, prevalence shift of 0.2, sample

size 200 (equivalent to the results shown in Figure 3c). Even though inclusion of the

confounding variable in the model formula improved the precision of corncob compared to

the “naive” implementation, corncob’s confounder-adjusted precision was still much worse

than the theoretically expected value of 0.95 and for moderately strong confounding (φ=0.4)

even worse than the other method’s precision for their “naive” implementation. In short, the

issues with precision and AUROC we had observed for corncob in the non-confounded

benchmark also appeared in the confounded benchmark.

Actions taken:

We clarified the inclusion and exclusion of methods for the confounded benchmark in the

revised manuscript (page 9, lines 323-327).

Reviewer 2

Wirbel et al have reviewed several prior simulation / benchmarking studies on WGS and 16S

DA methods in a comprehensive report, "A realistic benchmark for the identification of

differentially abundant taxa in (confounded) human microbiome studies". This reviewer

believes the claimed uniqueness to their study is in the claim that 'previous DA benchmarks

relying on simulated datasets did not quantitatively evaluate the similarity to real data, which

undermines their recommendations'. Leveraging that premise, they shift, scale, sample etc.

from existing datasets to and then evaluate common methods. There are several major

concerns that I believe can be addressed; however, previous reports have already

performed simulations on 'existing' datasets perturbed or randomly sampled with shifts

leading to similar results.

We thank the reviewer for their critical assessment of our manuscript and the very

constructive feedback.

Major concerns:

1. Quite a few benchmarking studies that have been performed in the past. This

includes leveraging very similar approaches to that of Wirbel et al. The novelty in assessing

the PCo coordinates of 'real', 'simulation / perturbation', etc. is unique. However, the

reviewer has a difficulty in reviewing prior benchmarking studies assessed and is aware of

one in particular that has randomly sampled existing study samples and assigned novel case

status and scaled abundances.

a. Some of the more simple benchmarking studies have occurred as far back as 2016

where samples underwent the same process as described in Wirbel et al

https://link.springer.com/article/10.1186/s40168-016-0208-8#Fig1

b. I am not suggesting a multi-'true' cohort (think n-dimensional comparison with

multiple underlying studies, ie. Not just the zeevi et al study), but I would include at a

minimum a table of all prior benchmarking studies and their approaches.

Response:

We appreciate this feedback from the reviewer, as well as the citation of Thorsen et al.,

which we had regrettably overlooked in our original manuscript but is now included.

Indeed the approach of Thorsen et al. is similar to ours in its use of additive and/or

multiplicative spike-in effects to generate differential abundance effects. However, an

important difference is that they generated signals in only one of their two randomly

determined groups before rescaling samples to their original counts. This is practically

identical to the implementation we carried out to examine the impact of compositionality on

DA method behavior. Our results strongly suggest that this approach, as expected, induces

unwanted compositional effects (also discussed in our response to Reviewer 1’s Point 1),

which is problematic for benchmarking DA methods.

In particular we appreciate the reviewer’s suggestion to include tables that provide a better

overview of the somewhat crowded and diverse DA benchmark landscape. We have now

included two supplementary tables comparing what we would consider some key attributes

of both DA benchmarks / simulation strategies, and DA methods covered in our study.

Actions taken:

We included a reference to Thorsen et al. in the revised manuscript (page 4, line 152). We

additionally added Supplementary Tables 1 and 2, which provide an overview of previous

simulation and benchmarking studies, respectively.

2. Missing methods:

a. While Wirbel et al have cited the biorxiv paper of Mallick H et al (MaAslin2) and used

several methods implemented within the R package. The paper has however, was

accepted in 2021 and has over 700 google citations. This reviewer would appreciate

the inclusion of the primary Maaslin2 method for comparison.

b. Personally, I believe the standard t-test to be robust, but this method is catching the

microbiome community by storm and is relevant enough to compare to.

Response:

We appreciate the reviewer’s feedback and suggestions here and apologize for this outdated

citation. With regard to Point 2a, we have updated the citation and added Mallick et al. to our

new Supplementary Table 1. We would like to clarify that the TSS-normalized and log-

transformed linear model(s) in our benchmark (i.e. the LM as described in e.g. main text Fig.

2) are identical to the default settings of MaAsLin2, as specified in the main text of their

manuscript and codebase on GitHub.

From Mallick et al., “While the default MaAsLin2 implementation uses a log-transformed

linear model on TSS-normalized quality-controlled data, the software supports several other

statistical models including count models” (e.g. the negative binomial GLM, which we also

tested). Since other tools make use of the same pre-processing and base R linear model in

their differential abundance testing framework (e.g. the updated SIAMCAT R package

developed in our group) and because it is not the only implementation MaAsLin2 offers, we

thought it best to use the broader term LM. For the confounded simulations we also chose to

implement the lmerTest::lmer() mixed effects model over the also-popular lme4::lmer()

method to specifically improve comparability with MaAsLin2.

Since readers may share the reviewer’s concern, given the popularity of MaAsLin2, we have

more explicitly pointed out in our manuscript that we replicate the MaAsLin2 defaults. We

moreover verified that our implementation behaved as expected (demonstrated below,

showing P-values derived from our implementation of the linear model and using the

MaAsLin2 tool on our simulations), and this is now mentioned in our revised manuscript.

6

With regard to Point 2b, we thank the reviewer for their suggestion. After adding the

Student’s t-test to our benchmarks (implemented with defaults as t.test in base R), we found

that it outperformed a majority of parametric and microbiome-specific methods (e.g. ANCOM

tools), analogous to other classical statistical methods we included. As a result, we updated

all main and supplementary figures showing the results of the benchmark (e.g. Fig. 2) and

the main text to reflect this more general performance trend.

Actions taken:

We included MaAsLin2 in our new Supplementary Table 1. We also specified that our

implementation of the linear model mirrors and exactly replicates the default settings in

MaAsLin2 in the Methods and Discussion (page 14, lines 510-511 and page 18, line 679-

680). Additionally, we added the t-test to the non-confounded benchmark. We updated all

relevant figures (Fig. 2, SFigs. 5-9,15) and made several changes to the text (e.g. page 7,

lines 243-244) as well as the Abstract (page 2, lines 59-60).

3. The authors do not provide enough evidence in the discussion surrounding their

claims that, 'Based on our results, we argue that the failure to produce consensus is partially

explained by their reliance on unvalidated and ultimately unrealistic parametric simulations of

microbiome data as ground truth.'

a. Authors have not performed an exhaustive analysis of similar disease cohorts and

assessed the heterogeneity in raw abundances. There exist many meta-analyses,

e.g. https://www.nature.com/articles/s41467-017-01973-8 that have adequately come

to conclusions of relevant organisms implicated in disease

Response:

We appreciate the reviewer’s feedback and the chance to clarify our writing, which we think

may have been misunderstood in its original context. We agree with the reviewer (and the

authors of the referenced publication, ref. 9 in our original submission) that it is possible to

achieve consensus in terms of biological results (i.e., which microbial organisms might be

related to a disease). Indeed, our group has previously published on microbiome meta-

analyses, for example for colorectal cancer, and we believe that a consensus concerning

colorectal cancer-associated bacteria has emerged in the field – also given that meta-analyses by other groups resulted in very similar results (see

https://www.nature.com/articles/s41591-019-0406-6 and

https://www.nature.com/articles/s41591-019-0405-7).Of course, the DA methodology for

meta-analyses needs to be validated as well, as a meta-analysis relying on a DA tool that

produces an excess of false positive discoveries will be methodologically flawed as well.

The claim in question intended to highlight the lack of consensus among DA benchmarks,

meaning that there is no consensus on which DA methodology is best-performing for

microbiome data. We argue (and we believe we show) that this is partially due to

heterogeneity among the (unvalidated) parametric simulation strategies employed earlier

(see page 8, lines 272-281 and Fig. 2d).

Actions taken:

We updated the sentence in question in our Discussion (page 13, lines 450-453) to improve

clarity for readers. Additionally, we highlighted that meta-analyses (with validated

methodology) are good tools to overcome discrepant findings from individual studies (page

14, lines 517-523) provided that they are based on valid statistical methods.

4. The inclusion of the methods scripts is nice, but for true broad generalizability and

impact / use the authors should create a sustainable Bioconductor (at a minimum CRAN) R

package like sparsedossa or https://www.ncbi.nlm.nih.gov/pmc/articles/PMC7047923/

Response:

We agree with the reviewer and thank them for the comment. We have submitted SIMBA to

CRAN, as it was already formatted as an R package (and could be installed from GitHub

using the devtools package). The BAMBI part of the project is a collection of scripts that

allow for the execution of several cluster jobs that make use of SIMBA functions and is by its

nature not an R package, but rather a set of scripts that we used in this project. For

reproducibility, the exact same environment as used in our project can be re-created from

renv.lock file included in the BAMBI project.

Actions taken:

We have submitted SIMBA to CRAN and have specified this in our revised methods (page

22, line 902).

Reviewer 3

This is a well written and interesting manuscript. It contains two important contributions: (1) a

new simulation framework for simulating realistic microbiome data, and (2) a benchmark of

many DA methods. Moreover, it also evaluates methods in the presence of confounding

effects.

Response:

We would like to thank the reviewer sincerely for their thoughtful consideration of our work,

and especially for their polite, constructive communication.

Comments:

1. There is one more microbiome simulation method that I am aware of, and which also

succeeds well in simulating realistic data. SPSimSeq (Assefa et al., 2020, 2023) was

originally developed for simulating (bulk) RNASeq data, but it has been demonstrated to

work also well for microbiome data (16S rRNA sequencing); see Kodalci and Thas (2023).

Response:

We appreciate the reviewer bringing this interesting simulation framework to our attention.

We agree with the reviewer that SPSimSeq managed to simulate quite realistic microbiome

data. We applied it to the Zeevi WGS dataset and simulated a dataset without any signal.

We evaluated the realism in a similar way to the other simulation methods included in Fig. 1

in the main text (see Figure below). The feature variance (panel a) values very closely mimic

those of real data; sample sparsity (panel b) from SPSimSeq was also fairly similar to real

data. Likewise, the PCoA shows the real and simulated data overlapping (panel c). A

machine learning-based comparison resulted in an AUROC of 1, but this was due to

SPSimSeq reordering and randomly resampling features for simulation, so that a direct

feature-aware comparison was impossible.

If we understood correctly, for its use in benchmarking DA tests, SPSimSeq requires the

input data to have a biological signal present (i.e. differential gene expression between two

measured conditions, and extensible to differential microbial abundances), which is first

identified (via a linear model) and then used to simulate either background features (based

on those without differential expression detected) or differentially abundant features (based

on those features identified in the first step). This is complementary to our approach, which

by necessity takes input data without differential biological signal before implanting fully

synthetic effects.

The complementary nature of the two approaches made it difficult to include SPSimSeq in

the comparison of simulation frameworks, because our approach was intentionally based on

datasets without biological signal (e.g. Zeevi et al., Schirmer et al., or the HMP datasets) to

be able to flexibly control the implanted effect sizes. We therefore did not include SPSimSeq

in our benchmark of simulation approach, but instead refer to it in the main text and the new

Supplementary Tables.

Actions taken:

We added a references to Kodalci and Thas to the Discussion (see page 14, lines 506-508)

as well as our new Supplementary Table 1, which also references SPsimSeq (Assefa et

al.).

2. For the performance evaluation you compare the empirical FDR with the estimated FDR.

What is the estimated FDR?

Response:

We appreciate this feedback from the reviewer and welcome the opportunity to clarify this.

The present terminology was introduced in an earlier revision, and we agree it could be more

intuitive. In our manuscript, the estimated FDR is the result of applying a multiple hypothesis

testing correction procedure to the P values obtained from the DA testing methods, and thus

represents the expected proportion of false positives among all discoveries based on the

method’s assumptions. The empirical FDR, in contrast, is observed in our benchmark (i.e.

calculated using the simulated ground truth), and represents the actual proportion of false

positives among all discoveries made by a method at a given estimated FDR cutoff.

Actions taken:

We have replaced “empirical” with “observed” everywhere in the manuscript to make the

distinction between these concepts clearer for readers (page 6, lines 214-225).

3. Lin and Peddada (2020) argue that microbiome data often shows a differential sampling

fraction between the two groups. Their method (ANCOM-BS), which is also included in your

study, is supposed to adjust for it. Does your simulation method allow for the introduction of

differential sampling fractions? A related issue: the library size distribution may be different

between the two groups. If this happens, then it is either by coincidence, or because of

technical issues. Anyway, it may affect the DA results. Your simulation method starts with

randomly splitting the set of all samples into two groups, and hence, on average, no

difference between the sampling distributions is expected. Would it be possible to introduce

this flexibility in the R implementation? I am not necessarily suggesting that you need to

extend your simulation study in this way, but it could be added to the discussion section.

Response:

We appreciate the reviewer’s perspective on this issue – this is indeed an interesting aspect.

In fact, our simulation method does allow for the introduction of differential sampling

fractions.

While this wasn’t a major focus of our simulation studies, by implanting signal into only one

of our two random groups and then rescaling samples to their original library sizes, we were

showed, that due to compositionality, secondary differential abundances are introduced in

the background taxa (see SFig. 9, also our responses to Point 1 by Reviewers 1 and 2). If

the rescaling step were to be removed, it would be analogous to what the reviewer

describes. Additionally, for follow-up studies aiming to explore this further, it would of course

be easily possible to downsample reads from one of the groups (to simulate library size

differences) after feature implantation.

Actions taken:

We updated the Methods section (page 17, lines 643-644) and the Discussion section (page

14, lines 495-497) to point out this possibility.

4. In the Methods section you give a clear description of the simulation method. It is

mentioned that after scaling and shifting, the generalised fold change is computed for all taxa, and if a fold change for an implanted feature is smaller than 0.001, then that feature is

removed. This makes sense to me, but what about the other way around? Suppose that

after randomly splitting samples into two groups, a non-DA feature shows – by coincidence –

a large fold change?

Response:

We thank the reviewer for this comment. We investigated to which extent background

features might have larger fold change than the implemented features. We calculated the

AUROC for distinguishing implanted from background features based on the gFC across

different effect sizes. Except for the very low effect sizes, we observed an almost perfect

separation between implanted and background features as shown below, indicating that

implanted signals are consistently above the background. We added this plot as panel b to

SFig. 4 in the revised manuscript and highlighted the effect size that is chosen as an

example in the main figure panels.

Actions taken:

We added the panel of AUROC values for distinguishing implanted and background features

based on the generalized fold change to SFig. 4 as an additional panel. We furthermore

changed the text of the revised Methods section (page 17, lines 634-637).

5. Whereas the description of the simulation method in the absence of confounders is very

clear to me, this does not apply to the description of the generation of confounding effects (p.

21). Although I think I can more or less what you did, I am afraid that the description is

insufficiently clear for most people to reproduce the simulations, and reproducibility is

important. Can you please try to provide a better description?

Response:

We agree with the reviewer and thank them for this important feedback and opportunity.

Actions taken:

We have rewritten both the Results and Methods sections discussing our confounded

simulations (page 9, lines 312-329 and page 20, lines 795-812, respectively). We

streamlined the Results by focusing only on the simulations that mimic “medication”-like

confounder effects to reduce the possibility of confusing our readers.

6. With the linear models, I wonder why you have chosen to adjust for confounders by

adding them as random effects to the model? Don’t most people add the confounder as a fixed effect to the model? For the Wilcoxon rank sum test, you have chosen for stratification

if I am correct. This is more like considering the confounder as a fixed effect. By the way,

note that Kodalci and Thas (2023) proposed extensions of the Wilcoxon rank sum test that

allows for the adjustment of continuous confounders/covariates.

Response:

We appreciate the reviewer’s insights here, and the overlooked reference to Kodalci and

Thas. The reviewer is correct that we have chosen a stratified Wilcoxon test (from the coin

package), which is similar to a fixed confounder effect in a linear model (when that

confounder is a binary variable); however, the stratified Wilcoxon also uses permutations.

Regarding the choice of random confounder effects, we concur this is somewhat atypical for

binary confounders in simple case-control studies. However, we opted to test this

implementation due to its scalability to more complex study designs, and potential statistical

advantages in handling unbalanced and correlated observations (which we consider an

important aspect of real clinical microbiome data). We added a performance evaluation of a

fixed effects model in comparison, and observed it to be largely the same on our simulated

data (see SFig. 11).

Actions taken:

We added a reference to Kodalci and Thas in the Discussion where we discuss our

limitations and scope (page 14, lines 506-508). We also clarified the text outlining our

choices in both the Methods (page 18, lines 678-686), Results (page 9, lines 323-329), and

added references to SFig. 11 on page 9.

7. When using linear models, the confounders are added as additive, linear effects.

Does this agree with the way the data is simulated? For the real data analyses, you

could e.g. make residual plots to check this assumption.

Response:

We appreciate the reviewer for raising the point about alignment between our data

simulation approach and the assumptions of linear models. For the simulated data, we

implanted signals for the main variable and confounder independently into different (non-

overlapping) random subsets of features (10% each), which were then subjected to a

separate (biased) resampling process that prioritised certain combinations of the main

variable and confounder based on a predefined probability. Our previous analysis showing a

similar performance of different linear model formulae provides some evidence that our

resampling did not introduce non-linear or interaction effects in the ground truth of our

confounded simulations (see SFig. 11).

We also appreciate the suggestion regarding model diagnostics for our real data analyses.

Below we provide some context for the taxa identified in the main text Fig. 4a. This extreme

but not uncommon biological case whereby there is clear non-independence (i.e. hierarchy)

between case-control status and confounder variable (here, diabetes and metformin intake)

also motivated our implementation of a random confounder effect.

In a we have identified the “best” and “worst” fitting models based on the assumption of

homoscedasticity: E. lenta and B. crossotus, respectively. B. crossotus was identified as disease-associated, likely due to the increased prevalence in the control group. In d-g we

show the raw abundances (from quantitative microbiome profiled data, i.e. absolute cell-

count adjusted values), including R. gnavus and I. bartlettii for further context. In the main

text Fig. 4, we identified these (respectively) to be strongly enriched in diabetics (regardless

of metformin status) and strongly depleted in metformin-taking diabetics exclusively. In h-k,

we show the fitted group means and residual values.

We also created an additional supplementary figure similar to the one shown here but for our

simulated data, where we explored the assumptions of linear models specifically for ground

truth features. Overall, we concluded that while many taxa violated model assumptions, even

in the “worst” case linear mixed-effects models are a robust and practical solution for the

constraints of high-throughput DA testing scenarios. However, we would greatly appreciate

any constructive feedback on our new supplementary analysis and figure (SFig. 13) that the

reviewer is willing to provide.

Actions taken:

We clarified how we generated our confounded simulations in the Results (page 9, lines

312-322) and Methods (page 20, lines 795-812). We added an additional supplementary

figure similar to the one shown here examining linear (mixed-effect) model assumptions on

our simulated data (see SFig. 13).

8. Throughout the manuscript you use the term “post hoc” adjustment. I have doubts

about this terminology. Post hoc seems to suggest that the analysis is done in a second-

stage, after having seen the results of the first-stage data analysis. For example, in a

classical ANOVA, multiple comparisons of means is sometimes referred to as post hoc

testing, because in the first stage the ANOVA F-test indicated an effect of the factor variable.

We appreciate this feedback from the reviewer, and agree with their points. Instead, we

believe that the terminology “stratified DA testing” might be more appropriate to our

particular benchmark scenarios which deal exclusively with binary confounder variables.

Actions taken:

In all mentioned instances, we have either removed “post hoc” as an adjective for

“adjustment”, or replaced “post hoc adjustment” with “stratified DA testing”, depending on

context.

References

Assefa, A. T., Vandesompele, J., & Thas, O. (2020). SPsimSeq: semi-parametric simulation

of bulk and single-cell RNA-sequencing data. Bioinformatics, 36(10), 3276-3278.

Assefa A, Hawinkel S, Vandesompele J, Thas O, R Core Team (2023). SPsimSeq: Semi-

parametric simulation tool for bulk and single-cell RNA sequencing data. R package version

1.12.0.

Kodalci, L., & Thas, O. (2023). Simple and flexible sign and rank-based methods for testing

for differential abundance in microbiome studies. Plos one, 18(9), e0292055.

Lin, H., & Peddada, S. D. (2020). Analysis of compositions of microbiomes with bias

correction. Nature communications, 11(1), 3514.

**2nd round**

**Reviewer 1**

I greatly appreciate your attention to the comments that were made by myself and the other reviewers, thank you.

**Reviewer 2**

I thank the authors for their kind feedback and diligent efforts in addressing my comments. The paper is of higher quality than before with the inclusion of the t-test confirming previously benchmarks and papers. I apologize for not bringing this comment in earlier in the prior round, but in further review realized I was remiss to discuss one particular argument still discussed within the community.

Major comments:
- Compositionality of microbiome cohorts:

The authors write on L291, "... it is not disputed that sequencing produces compositional data...". I would suggest to the authors that there is indeed still great dispute as to whether microbiome data is fully compositional or a generative process that along with multiple 16S within specific organisms and potential preferential sequencing leads to greater abundances of certain sequences.

Minor comments:

Thank you for submitting the package to CRAN. Was there a reason the authors chose not to submit to Bioconductor? The level is much higher

**Reviewer 3**

The authors did a splendid job in reviewing the manuscript. They gave more than satisfactory answers to my questions. In their rebuttal letter they gave many details and they actually started interesting discussion that would be interesting to follow up upon.

Minor comments:
- The “estimated FDR” is still not well described/defined in the manuscript. My best guess is that it is the adjusted p-value (with e.g. Benjamini-Hochberg)? This comment does not jeoperdise the quality of the manuscript, but it would be good to make sure that it is clear to all readers.
- p. 8. There is one instance that still mentions “empirical FDR” instead of “observed FDR”.


As a final thought I want to mention that in studies like this reviewers can always ask to include more scenarios, and the eventual set of scenarios thus depends on both the authors and the reviewers. You can argue that it’s never enough scenarios that are included. However, at some point researchers need to stop and publish their results. To my opinion the authors succeeded very well in performing a neutral comparison of methods, under a sufficiently wide range of scenarios. They published their R code so that other researchers can use their simulation set-up, starting from other datasets and considering other scenarios. The conclusions of the authors are clear and robust.

**Authors’ response**

We modified the sentence on page 6 which introduces the estimated FDR to make the

statement clearer. Additionally, we modified the sentence about the compositionality of

metagenomics data on page 9.
